# Noise schemas aid hearing in noise

**DOI:** 10.1073/pnas.2408995121

**Published:** 2024-11-15

**Authors:** Jarrod M. Hicks, Josh H. McDermott

**Affiliations:** ^a^Department of Brain and Cognitive Sciences, Massachusetts Institute of Technology, Cambridge, MA 02139; ^b^McGovern Institute, Massachusetts Institute of Technology, Cambridge, MA 02139; ^c^Center for Brains Minds and Machines, Massachusetts Institute of Technology, Cambridge, MA 02139; ^d^Program in Speech and Hearing Bioscience and Technology, Harvard University, Boston, MA 02115

**Keywords:** auditory scene analysis, hearing in noise, sound texture

## Abstract

Human hearing is robust to noise, but the basis of this robustness is poorly understood. Several lines of evidence are consistent with the idea that the auditory system adapts to sound components that are stable over time, potentially achieving noise robustness by suppressing noise-like signals. Yet background noise often provides behaviorally relevant information about the environment and thus seems unlikely to be completely discarded by the auditory system. Motivated by this observation, we explored whether noise robustness might instead be mediated by internal models of noise structure that could facilitate the separation of background noise from other sounds. We found that detection, recognition, and localization in real-world background noise were better for foreground sounds positioned later in a noise excerpt, with performance improving over the initial second of exposure to a noise. These results are consistent with both adaptation-based and model-based accounts (adaptation increases over time and online noise estimation should benefit from acquiring more samples). However, performance was also robust to interruptions in the background noise and was enhanced for intermittently recurring backgrounds, neither of which would be expected from known forms of adaptation. Additionally, the performance benefit observed for foreground sounds occurring later within a noise excerpt was reduced for recurring noises, suggesting that a noise representation is built up during exposure to a new background noise and then maintained in memory. These findings suggest that noise robustness is supported by internal models—“noise schemas”—that are rapidly estimated, stored over time, and used to estimate other concurrent sounds.

## Significance

Noise is ubiquitous in the auditory world, and human hearing is remarkably robust to its presence. The standard explanation for this robustness involves adaptation to components of the auditory input that are stable over time, accentuating time-varying signals at the expense of static “noise” signals. Here, we show several properties of human noise robustness that are inconsistent with such a simple explanation. The results are instead consistent with the idea that the auditory system estimates the properties of noises it encounters and then stores them over time, using the resulting internal model to estimate other concurrent sounds.

Much of the everyday listening experience is distorted by noise. Although noisy environments present a challenge for hearing, human listening abilities are remarkably robust to noise, enabling us to converse over the hum of a restaurant or recognize sounds on a windy day. However, the ability to hear in noise is vulnerable, declining with age (1, 2) and following hearing loss (1, 3). Understanding the basis of noise-robust hearing and its malfunction is thus a central goal of auditory research.

Noise robustness has been well documented in humans. For instance, speech intelligibility falls off gradually with signal-to-noise ratio (SNR), but remains high even when background noise has comparable power to a concurrent speech signal (4). Additionally, some types of sounds are easier to hear in noise than others (5), and hearing is more robust to some types of noise than others (6–10). Moreover, neural correlates to this robustness have been discovered along the ascending auditory pathway of multiple species (11–20). Yet, despite recent interest in the factors that enable and constrain hearing in noise, the problem is not well understood in computational terms.

**Fig. 1.**
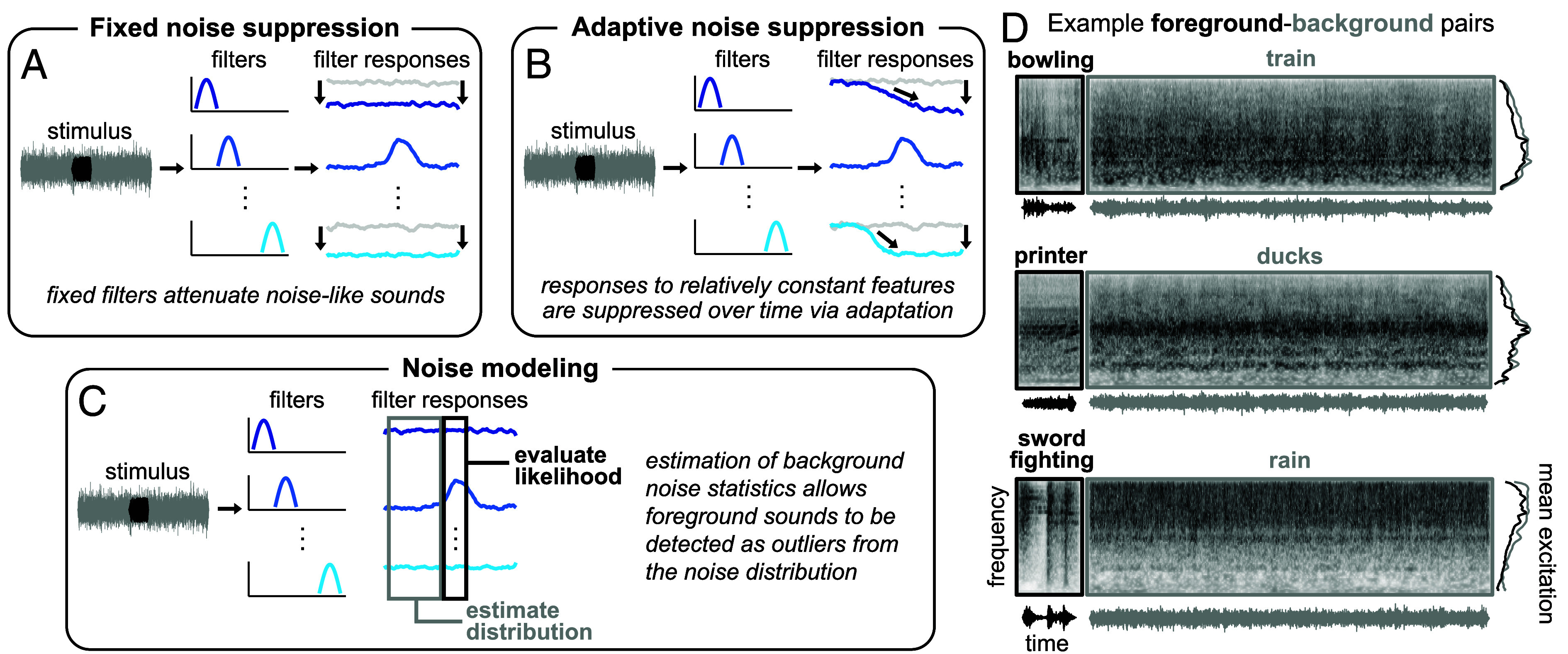
Potential explanations for noise-robust hearing and examples of experimental stimuli. (*A*) Fixed noise suppression hypothesis. A stimulus waveform (*Left*, background shown in gray, foreground shown in black) passes through a set of filters (*Middle*, transfer functions shown in shades of blue for an unspecified stimulus dimension), resulting in a set of filter responses over time (*Right*). Here and in (*B* and *C*), the filter tuning is unspecified and is not essential to the general predictions of the hypotheses. The filters could be nonlinear functions of the input and might measure higher-order properties of sound. In the fixed noise suppression hypothesis, the gain of a fixed set of filters is reduced to attenuate noise-like sounds (light gray responses show unattenuated filter response). (*B*) Adaptive noise suppression hypothesis. In the adaptive noise suppression hypothesis, responses to relatively constant features are suppressed over time via adaptation. (*C*) Noise modeling hypothesis. In the noise modeling hypothesis, estimation of background noise statistics allows foreground sounds to be detected as outliers from the associated distribution. (*D*) Example sounds used to generate experimental stimuli. Each panel shows the foreground (*Left*, black) and background (*Right*, gray) sound from an example trial, displayed as sound waveforms (*Bottom*), cochleagrams (*Top*), and mean excitation patterns (*Right*). Cochleagrams were generated from the envelopes of a set of bandpass filters with tuning modeled on the human ear. Darker gray denotes higher intensity. Mean excitation patterns were obtained by averaging the cochleagram over time. In our initial experiments, foreground–background pairs were selected to have similar long-term spectra to minimize differences in the spectrotemporal overlap that would otherwise cause large variation in detectability from across pairs. This design choice turned out not to be essential and was dropped in later experiments.

A common view is that the auditory system filters out or suppresses noise in order to recognize sources of interest. One possibility is that the brain has internalized typical properties of noise and, by default, suppresses them relative to the properties of other sound sources (9, 12, 17). For instance, because noise is often approximately stationary (i.e., being defined by statistical properties that are relatively constant over time), the auditory system could preferentially suppress stationary sounds, which might enable more robust recognition of other sounds in noise. We refer to this hypothesis as “fixed noise suppression,” the idea being that there are fixed filters that attenuate noise-like sounds (Fig. 1*A*). Another possibility is that noise properties are implicitly detected and suppressed via local adaptation mechanisms that reduce the response to features that are relatively constant in the auditory input (13, 14, 16, 18, 21–23). We refer to this hypothesis as “adaptive noise suppression” (Fig. 1*B*). While adaptive mechanisms can account for some of the observed neural responses to stimuli in noise, such proposals have primarily been evaluated with simple synthetic noise signals, leaving it unclear whether they explain robustness to noise sources containing the rich statistical structure present in natural environments (24).

An alternative possibility is that the auditory system might actively model the statistical structure of noise (Fig. 1*C*). This idea derives some a priori plausibility from the potential of background noise to convey useful information. Although laboratory studies of hearing in noise tend to use a single type of unstructured synthetic noise (e.g., white noise or pink noise), “noise” in the world can vary dramatically from place to place, often providing behaviorally relevant information about the environment (25, 26), such as the intensity of rain or wind. Such real-world noises are commonly referred to as auditory textures (24, 27). Auditory textures are typically generated by superpositions of many similar acoustic events and can exhibit a diversity of statistical properties (24). Moreover, human listeners are sensitive to statistical regularities of textures (24, 28–32) and estimate and represent their properties even in the presence of other sounds (33, 34). These considerations raise the possibility that rather than simply suppressing noise, the auditory system might model its statistical structure, using the resulting model to aid the separation of noise from other sound sources akin to how “schemas” are thought to aid the segregation of familiar words and melodies (35–37). Thus, there are at least three potential explanations for noise-robust hearing: fixed noise suppression, adaptive noise suppression, and the internal modeling of noise schemas.

We sought to test these three candidate explanations for noise-robust hearing and assess their role in everyday hearing. Adaptive suppression and internal noise modeling both predict that the ability to hear in noise should improve following the onset of a noise source: adaptation should grow over time, and a noise model should be more accurately estimated with larger samples. Such temporal effects have been documented in a few tasks (38) including pure tone detection (classically termed “overshoot”) (39, 40), amplitude modulation detection (41), phoneme recognition (18, 42), and word recognition (43, 44). However, because much of this work was conducted using relatively unstructured synthetic noise, it was unclear whether such temporal effects might be observed in more natural contexts (e.g., with realistic noise that is not fixed throughout a listening session). We thus began by characterizing listeners’ ability to detect, recognize, and localize natural foreground sounds embedded in real-world background noise.

Although adaptive suppression and internal noise modeling are not necessarily mutually exclusive (*Discussion*), they could be differentiated via the time course of their effects. Specifically, neural adaptation in the auditory system typically dissipates fairly rapidly following a stimulus offset (18) such that its effects would be expected to wash out during an interruption to background noise. By contrast, an internal model of noise properties might be maintained over time, yielding more persistent effects. Thus, to distinguish these two hypotheses, we further investigated whether noise robustness would persist across interruptions in noise and whether it might improve following intermittent repeated exposure to particular background noises. Such improvement would be expected if listeners learn noise schemas akin to the schemas acquired for melodies (37), but not if they simply adapt to ongoing noise in the environment.

We found that the ability to detect, recognize, and localize foreground sounds in noise improved over the initial second of exposure to the background, a timescale substantially longer than previously reported for synthetic noise and artificial tasks. We also found that foreground detection performance was robust to temporary changes in the background, suggesting that listeners maintain a representation of noise properties across interruptions. Moreover, detection performance was enhanced for recurring background noises, suggesting that internal models of noise properties—noise schemas—are built up and maintained over time. Finally, we found that the pattern of human performance could be explained by an observer model that estimates the statistics of ongoing background noise and detects foreground sounds as outliers from this distribution. Taken together, the results suggest that the predictable statistical structure of real-world background noise is represented in an internal model that is used by the auditory system to aid hearing in noise.

## Results

To characterize real-world hearing-in-noise abilities, we sourced a diverse set of 160 natural foreground sounds and background noises to create experimental stimuli. Each stimulus consisted of a brief foreground sound paired with an extended background noise. Foreground sounds were 0.5-s excerpts of recorded natural sounds (45, 46), and background noises were 3.25-s excerpts of sound textures synthesized (24) from the statistics of real-world textures drawn from a large set of YouTube soundtracks (AudioSet) (47, 48). In our initial experiments, foreground–background pairs (Fig. 1*D*) were selected to have similar long-term spectra to avoid large differences in spectrotemporal overlap that would otherwise cause large variation in detectability across pairs.

### Experiment 1: Foreground Detection Improves with Exposure to Background Noise.

**Fig. 2.**
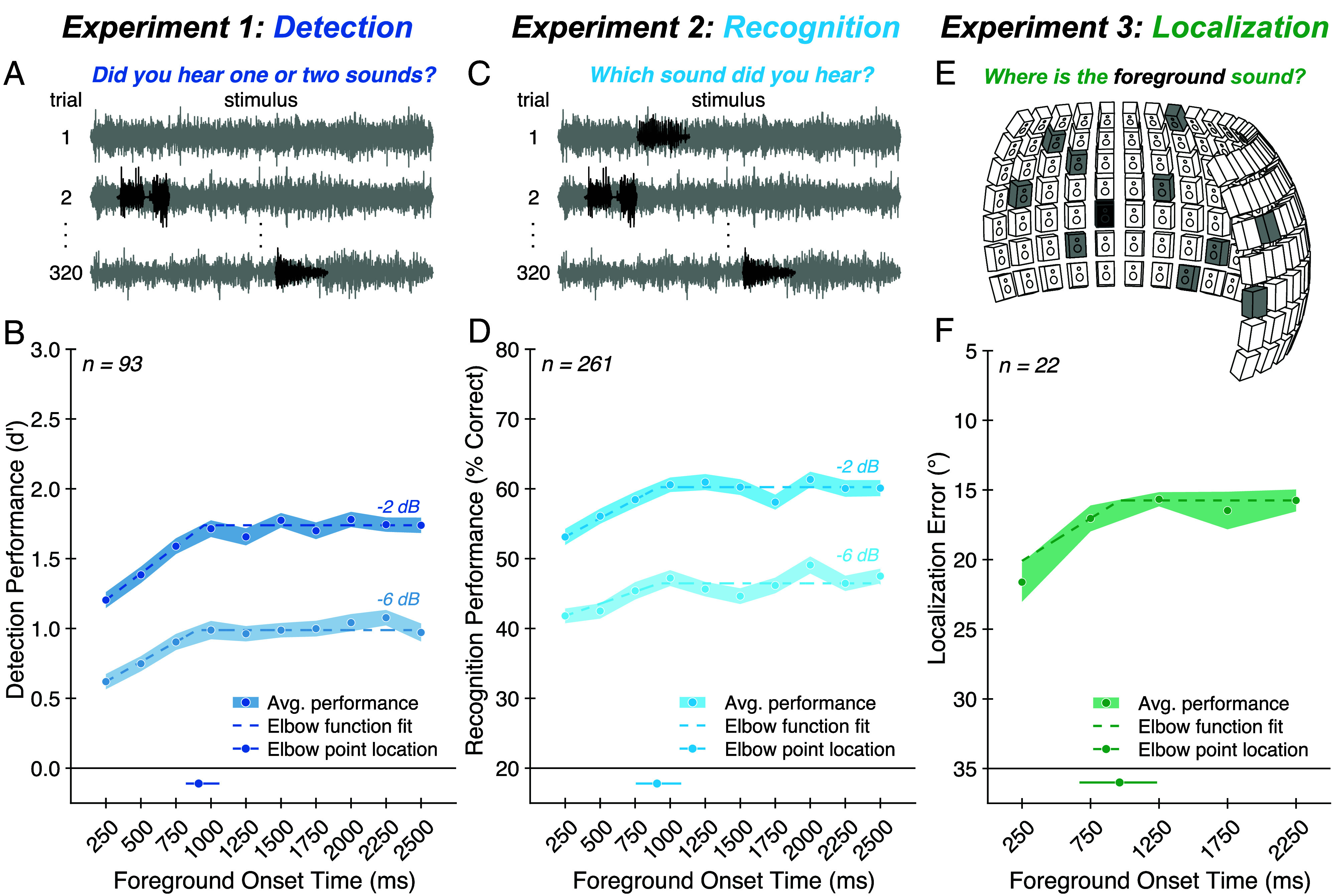
Experiments 1 to 3: Foreground detection, recognition, and localization improve with exposure to background noise. (*A*) Experiment 1 task. On each trial, participants heard a continuous background noise (gray) presented either in isolation (e.g., trial 1) or with a brief additional foreground sound (black) superimposed (e.g., trial 2). We manipulated the onset time and SNR of the foreground relative to the background. Participants judged whether the stimulus contained one or two sound sources. (*B*) Experiment 1 results. Average foreground detection performance (quantified as d′; blue circles) is plotted as a function of SNR and foreground onset time. Shaded regions plot SE. Dashed lines plot elbow function fit. The solid line below the main axis plots one SD above and below the median elbow point, obtained by fitting elbow functions to the results averaged over SNR and bootstrapping over participants; the dot on this line plots the fitted elbow point from the complete participant sample. (*C*) Experiment 2 task. On each trial, participants heard background noise (gray) containing a foreground sound (black) and were asked to identify the foreground by selecting a text label from five options. (*D*) Experiment 2 results. Foreground recognition performance (quantified as percent correct; blue circles) is plotted as a function of SNR and foreground onset time. Chance performance was 20%. Data are plotted using the same conventions as (*B*). (*E*) Experiment 3 task. Stimuli were presented via an array of 133 speakers spanning −90° to +90° in azimuth and −20° to +40° in elevation. On each trial, participants heard a scene composed of diffuse background noise (different samples of a texture played from 10 randomly selected speakers, shown in gray in the diagram) and a foreground sound (played from a randomly selected speaker, show in black in the diagram) occurring at one of five temporal positions within the noise. Participants judged the location of the foreground sound. (*F*) Experiment 3 results. Average foreground localization performance (quantified as absolute localization error in azimuth, in degrees; green circles) is plotted as a function of foreground onset time. The *y* axis is oriented to match conventions in other panels where higher positions along the ordinate indicate better performance. Data are plotted using the same conventions as (*B*).

We began by measuring the detection of natural sounds embedded in real-world background noises (Fig. 2*A*). On each trial, participants heard a continuous background noise presented either in isolation or with a brief “foreground” sound superimposed, then judged whether the stimulus contained one or two sound sources. Across trials, we manipulated both the temporal position and SNR of the foreground relative to the background. We chose the temporal positions so that there was always an onset asynchrony of at least 250 ms between foreground and background. Previously reported temporal dependencies of tone-in-noise detection (in which detection is better when tone and noise are asynchronous; "overshoot") are limited to asynchronies of less than a few hundred milliseconds (39, 40, 49). However, it seemed plausible that longer timescales might be evident with more naturalistic noise sources and foreground sounds.

Because task performance might benefit from knowledge of the foreground sounds (35–37), it was important that listeners only heard each foreground sound once during the experiment. To achieve this goal, we used an experimental design in which each participant completed only one trial for each of the 160 foreground–background pairings, with each pairing randomly assigned to one of the 20 experimental conditions (10 temporal positions crossed with 2 SNRs). Since this design necessitated a large sample size, we conducted this and other experiments online (with the exception of Experiment 3). Each of the 160 background noises also occurred once without a foreground sound. We calculated a single false-alarm rate from these background-only trials along with a hit rate for each of the 20 experimental conditions.

We found that foreground detection performance (quantified as d′) improved with exposure to the background [Fig. 2*B*; main effect of foreground onset time: F(9,828) = 22.85, *P* < 0.001, $\eta_{\mathrm{partial}}^{2}=\;0.20$]. As expected, we also saw better foreground detection performance at the higher SNR [main effect of SNR: F(1,92) = 769.15, *P* < 0.001, $\eta_{\mathrm{partial}}^{2}=\;0.89$], but the benefit of background exposure was evident at both SNRs [no significant interaction between foreground onset time and SNR: F(9,828) = 0.76, *P* = 0.65, $\eta_{\mathrm{partial}}^{2}=\;0.01$]. In both cases, task performance increased as the foreground sound was positioned later in the noise, with performance rising over roughly the initial second of exposure to the background.

This temporal dependence is consistent with the idea that listeners use the background noise preceding the foreground in order to perform the task. The temporal dependence also rules out several alternative possibilities. For example, if listeners performed the task entirely by detecting acoustic cues from the onset of the foreground sound, then task performance should be comparable at each temporal position of the foreground. Alternatively, if listeners could also perform the task equally well by listening retrospectively (using the background noise following the foreground to make a decision about the foreground′s presence), then performance should also be comparable across the different foreground positions since the total duration of background noise is the same for each condition.

To better quantify the timescale of the effect, we fit an “elbow” function (a piecewise linear function consisting of two line segments; *Materials and Methods*) to the results (averaged over SNRs). We bootstrapped over participants to obtain a CI around the location of the elbow point (i.e., the transition from rise to plateau). This analysis indicated that foreground detection performance improved with exposure to the background before reaching a plateau after 912 ms (95% CI: [812, 1,223] ms).

### Experiment 2: Exposure to Background Noise Benefits Sound Recognition.

In Experiment 2, we asked whether the benefit of background exposure extends to a recognition task. On each trial, participants heard a foreground–background pairing from Experiment 1 and were asked to identify the foreground by selecting a text label from five options (Fig. 2*C*). One option was the correct label; the remaining options were chosen randomly from the labels of the other foreground sounds in the stimulus set.

Recognition performance improved with exposure to the background in much the same way as did detection [Fig. 2*D*; main effect of foreground onset time: F(9,2340) = 9.04, *P* < 0.001, $\eta_{\mathrm{partial}}^{2}=\;0.03$; no significant interaction between foreground onset time and SNR: F(9,2340) = 0.84, *P* = 0.58, $\eta_{\mathrm{partial}}^{2}=\;0.00$]. The elbow function fit to these results indicated a timescale of improvement similar to that in the detection task from Experiment 1, with a plateau in performance after 905 ms (95% CI: [726, 1,242] ms) of exposure to the background.

### Experiment 3: Exposure to Background Noise Benefits Sound Localization.

We next asked whether the ability to localize sounds in noise similarly benefits from exposure to the background. We conducted this experiment in-lab using an array of speakers (Fig. 2*E*). On each trial, participants heard a scene composed of a foreground sound superimposed on spatially diffuse background noise, with the foreground occurring at one of five temporal positions within the background. Participants sat facing the array, holding their head still, and localized the foreground sound, entering the label of the corresponding speaker as their response. Because this experiment had to be run in person (rather than online), we chose to use only five temporal positions at a single SNR in order to reduce the total number of conditions, thereby increasing power and allowing us to collect data from a modest number of participants. Additionally, we lowered the SNR to account for the likelihood that spatial cues would reduce detection thresholds (50). It turned out that at the tested SNR, localization in elevation was close to chance. Thus, we quantified sound localization performance using the absolute localization error in azimuth only.

Sound localization improved with exposure to the background in a manner similar to that observed for detection and recognition tasks [Fig. 2*F*; main effect of foreground onset time: F(4,84) = 6.09, *P* < 0.001, $\eta_{\mathrm{partial}}^{2}=\;0.22$], with performance plateauing after 962 ms (95% CI: [750, 1,954] ms) of exposure to the background. Overall, the results point to a consistent benefit from noise exposure, spanning detection, recognition, and localization of natural sounds in noise.

### Experiment 4: Benefit of Background Exposure Persists Despite Knowing What to Listen for.

In real-world conditions, we often listen for particular sounds in an auditory scene. For example, when crossing the street, one might listen out for crosswalk signals, bike bells, or accelerating engines. Because expectations about a source can aid its segregation from a scene (35–37), and might also benefit hearing in noise, it was unclear whether the benefit of background exposure would persist if participants knew what to listen for. To address this issue, we conducted a variant of Experiment 1 in which participants were cued to listen for a particular foreground sound on each trial (*SI Appendix*, Fig. S1*A*). On each trial, participants first heard a foreground sound in isolation (the “cue”), followed by continuous background noise. Half of the trials contained the cued foreground sound superimposed somewhere on the background, and participants judged whether the cued sound was present. We again found that foreground detection performance improved with exposure to the background [*SI Appendix*, Fig. S1*B*; main effect of foreground onset time: F(9,1215) = 19.91, *P* < 0.001, $\eta_{\mathrm{partial}}^{2}=\;0.13$]. The timescale of improvement was similar to that in the detection task from Experiment 1, with a plateau in performance after 885 ms (95% CI: [582, 1,766] ms) of exposure to the background (not significantly different from the elbow point in Experiment 1; *P* = 0.52 via permutation test, elbow point difference: 68 ms). These results demonstrate that the benefit of background exposure persists even when participants know what to listen for, highlighting the relevance of this phenomenon for a range of real-world contexts.

### An Observer Model Based on Background Noise Estimation Replicates Human Results.

The results from Experiments 1 to 4 demonstrate a benefit of background noise exposure that provides evidence against the fixed noise suppression hypothesis, but that is conceptually consistent with both the adaptive suppression and internal noise modeling hypotheses. To first establish the plausibility of background noise estimation as an account of human hearing in noise, we built a signal-computable observer model to perform the foreground detection task from Experiment 1. The model evaluates the likelihood of incoming samples under a distribution whose parameters are estimated from past samples. The key idea is that samples not belonging to the background distribution (i.e., samples from the foreground sound) will tend to have low likelihood under a model of the background. Thus, the model can detect a foreground sound via samples that it assigns low likelihood. Intuitively, the fitted noise distribution should become more accurate with more samples, making it easier to detect outliers. However, it was not obvious that this type of model would achieve variation in performance with onset time that was on par with that observed in humans. We asked whether such a model could replicate the temporal dependence of foreground detection in noise observed in our human participants.

**Fig. 3.**
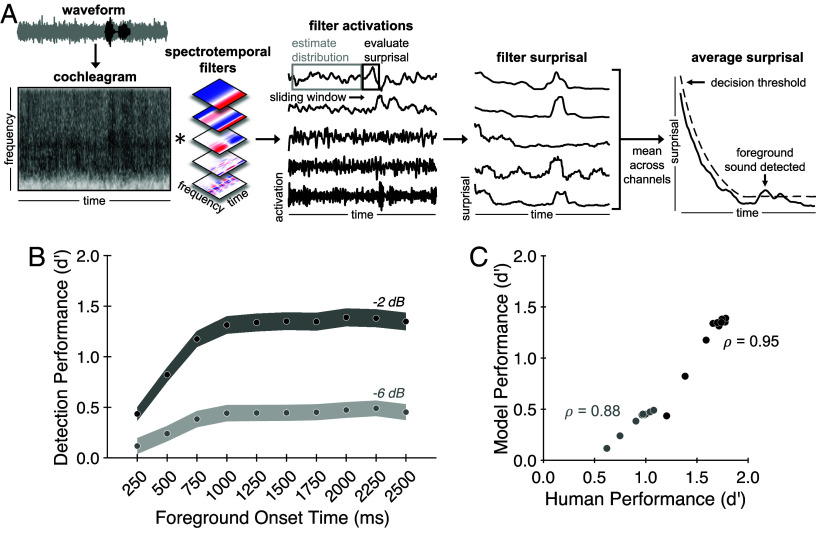
An observer model based on background noise estimation replicates human results. (*A*) Model schematic. First, an input sound waveform is passed through a standard model of auditory processing. This model consists of two stages: a peripheral stage modeled after the cochlea (yielding a cochleagram, first panel), followed by a set of spectrotemporal filters inspired by the auditory cortex that operate on the cochleagram, yielding time-varying activations of different spectrotemporal features (second panel). A sliding window is used to evaluate the negative log-likelihood (surprisal) within each filter channel over time (third panel). Finally, the resulting filter surprisal curves are averaged across channels and compared to a time-varying decision threshold to decide whether a foreground sound is present (fourth panel; *y*axis is scaled differently in third and fourth panels to accommodate the surprisal plots for multiple individual filters). (*B*) Model results. Model foreground detection performance (quantified as d′) is plotted as a function of SNR and foreground onset time. Shaded regions plot SD of performance obtained by bootstrapping over stimuli. (*C*) Human-model comparison. Model performance is highly correlated with human performance on the foreground detection task (Experiment 1) for both the −2 dB (black circles) and −6 dB (gray circles) SNR conditions.

A schematic of the model is shown in Fig. 3*A*. First, an input sound waveform is passed through a standard model of auditory processing consisting of two stages: a peripheral stage modeled after the cochlea (yielding a “cochleagram”), followed by a set of spectrotemporal filters inspired by the auditory cortex that operate on the cochleagram, yielding time-varying activations of different spectrotemporal features. Next, a probability distribution is estimated from these activations over a past time window and used to evaluate the negative log-likelihood of samples in a “present” time window. This quantity (“surprisal”) measures how unexpected the present samples are given the learned background distribution. The process is then stepped forward in time and repeated, resulting in a set of surprisal values for each spectrotemporal filter at each time point within the stimulus. The surprisal is then averaged across filter channels and compared to a decision threshold to decide whether a foreground sound is present. The decision threshold was determined empirically as a value of surprisal substantially greater than would be expected by chance (*Materials and Methods*).

For simplicity, we implemented the model with univariate normal distributions fit to each filter output as these were sufficient to account for the qualitative effects seen in human judgments. We note that this choice results in an impoverished model of sound texture. In particular, the distribution only models the mean and variance of filter activations while ignoring other higher-order statistics (e.g., correlations across filters) known to be important for sound texture perception (24). It also ignores temporal structure in the signal that exceeds the width of the filter kernels, treating all filter activations as independent. Although natural textures sometimes contain such high-order structure, it is at present unclear how this structure should be captured in a probabilistic model [existing models of texture (24) are based on a set of statistics rather than explicit probability distributions as are needed to evaluate outliers], and so we chose to sidestep this question to obtain a proof of concept for the general approach. The resulting simplifications would be expected to lower performance relative to what would be obtained with a distribution that more completely accounts for the statistical structure of natural textures. That said, the model captured some of the spectrotemporal structure of natural textures that differentiates them from traditional synthetic noise, and so seemed a reasonable choice with which to explore the general hypothesis of noise modeling.

The model was additionally defined by two hyperparameters: the width of the past window over which noise distribution parameters were estimated and the width of the present window over which surprisal was averaged. We tested a range of past and present window sizes and found that the best match to human data occurred with a past window size of 1,000 ms and a present window size of 500 ms. These results are presented here (see *SI Appendix*, Fig. S2 for the human-model correlation for different window sizes).

Despite its simplicity, the model qualitatively replicated the results from Experiment 1, showing a similar pattern of improvement with exposure to the background (Fig. 3*B*; see *SI Appendix*, Fig. S2 for model results with alternative window sizes, which remained qualitatively consistent with human results). Although model performance was below that of humans, the overall pattern of model performance across conditions was highly correlated with human performance (−2 dB SNR: ρ= 0.95, *P* < 0.001; −6 dB SNR: ρ= 0.88, *P* = 0.002). Overall, these results support background noise estimation as a plausible account of human hearing in noise by demonstrating that the qualitative trends evident in human behavioral performance can be explained by a model that estimates the statistics of ongoing background noise.

### Experiments 5a and 5b: Foreground Detection Is Robust to Background Interruptions.

**Fig. 4.**
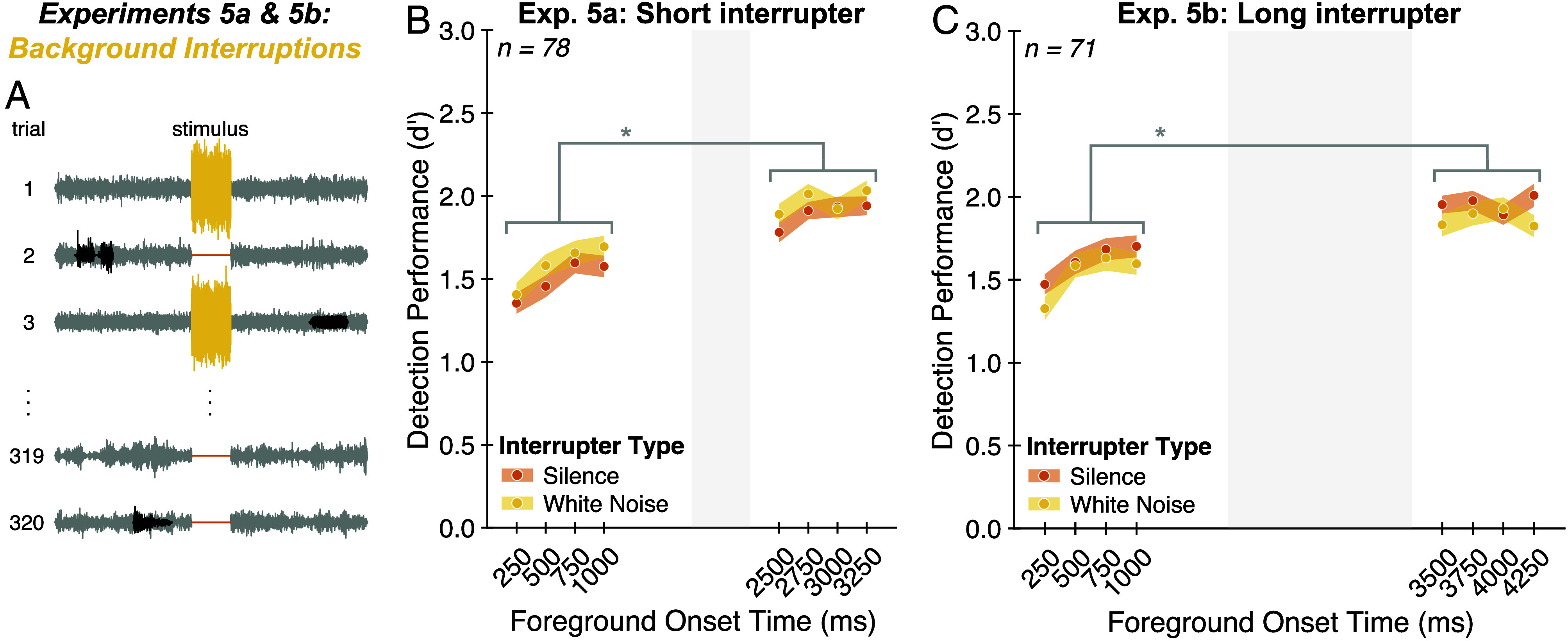
Experiments 5a and 5b: Foreground detection is robust to background interruptions. (*A*) Experimental task. Stimuli were like those from Experiment 1 but were modified by replacing the *Middle* 500 ms (Experiment 5a) or 1,500 ms (Experiment 5b) of background noise with either silence (orange) or white noise (yellow). Participants were asked to ignore this interruption and judge whether the stimulus contained one or two sound sources. (*B*) Experiment 5a results. Average foreground detection performance (quantified as d′) is plotted as a function of interrupter type and foreground onset time. Shaded regions plot SE. The gray region denotes the temporal position of interruption in background noise. * indicates statistical significance, *P* < 0.001. (*C*) Experiment 5b results. Same conventions as (*B*).

Experiments 5a and 5b aimed to distinguish the adaptive suppression and noise modeling hypotheses by testing the effect of interruptions to the background. We modified the stimuli from Experiment 1, temporarily interrupting the background noise with either silence or white noise, initially using a 500 ms interruption (Experiment 5a; Fig. 4*A*). The rationale was that this change to the background might cause adaptation to “reset,” leading to a decrement in foreground detection performance following the interruption. By contrast, the noise modeling hypothesis could allow for a benefit from background exposure despite the interruption, because the estimated noise parameters could be stored across the interruption.

Consistent with this latter possibility, foreground detection performance was greater for foregrounds following the interruption compared to those preceding the interruption [Fig. 4*B*; main effect of foreground position relative to interrupter: F(1,77) = 172.86, *P* < 0.001, $\eta_{\mathrm{partial}}^{2}=\;0.69$]. The pattern of results was similar for silent and noise interruptions [no significant interaction between interrupter type and foreground position relative to interrupter: F(1,77) = 0.12, *P* = 0.73, $\eta_{\mathrm{partial}}^{2}=\;0.00$].

To address the possibility that a 500 ms interruption was insufficient to trigger a complete release of adaptation (51), we ran an additional experiment (Experiment 5b) in which we increased the duration of the interrupter to 1,500 ms and asked whether the benefit of background exposure persisted. Despite the longer interruption, we again found that detection performance was greater for foregrounds following the interruption compared to those preceding the interruption [Fig. 4*C*; main effect of foreground position relative to interrupter: F(1,70) = 134.48, *P* < 0.001, $\eta_{\mathrm{partial}}^{2}=\;0.66$]. The pattern of results was again comparable for noise and silent interruptions [no significant interaction between interrupter type and foreground position: F(1,70) = 0.01, *P* = 0.92, $\eta_{\mathrm{partial}}^{2}=\;0.00$].

Perhaps the clearest evidence against an adaptation explanation is the fact that the results appear to not be affected by the duration of the interruption [500 versus 1,500 ms; no significant effect of interrupter duration when comparing performance for onset times after the interruption: F(1,147) = 0.08, *P* = 0.77, $\eta_{\mathrm{partial}}^{2}=\;0.00$]. Although the parameters of any adaptive processes that might be at play are not definitively established, one would almost surely expect a difference in release from adaptation for differences in interruption durations of this magnitude. Taken together, the results of Experiments 5a and 5b indicate that the benefit of background exposure is unlikely to reflect adaptation alone. Instead, listeners appear to maintain an internal representation of noise properties across temporary interruptions.

### Experiments 6 and 7: Repetition of Background Noise Enhances Foreground Detection.

We next investigated whether internal models of noise are built up over time, akin to the schemas that can be learned for recurring patterns in speech and music (36, 37). If listeners learn noise schemas and use them to aid hearing in noise, foreground detection should be enhanced for frequently recurring background noises. It also seemed plausible that the learning of a schema might reduce the “delay benefit”—the improvement in performance as the foreground onset is delayed relative to the noise onset—since listeners could use a stored representation of the noise properties, rather than having to estimate them online. We thus also tested whether the delay benefit would be altered if the noise repeated across trials.

**Fig. 5.**
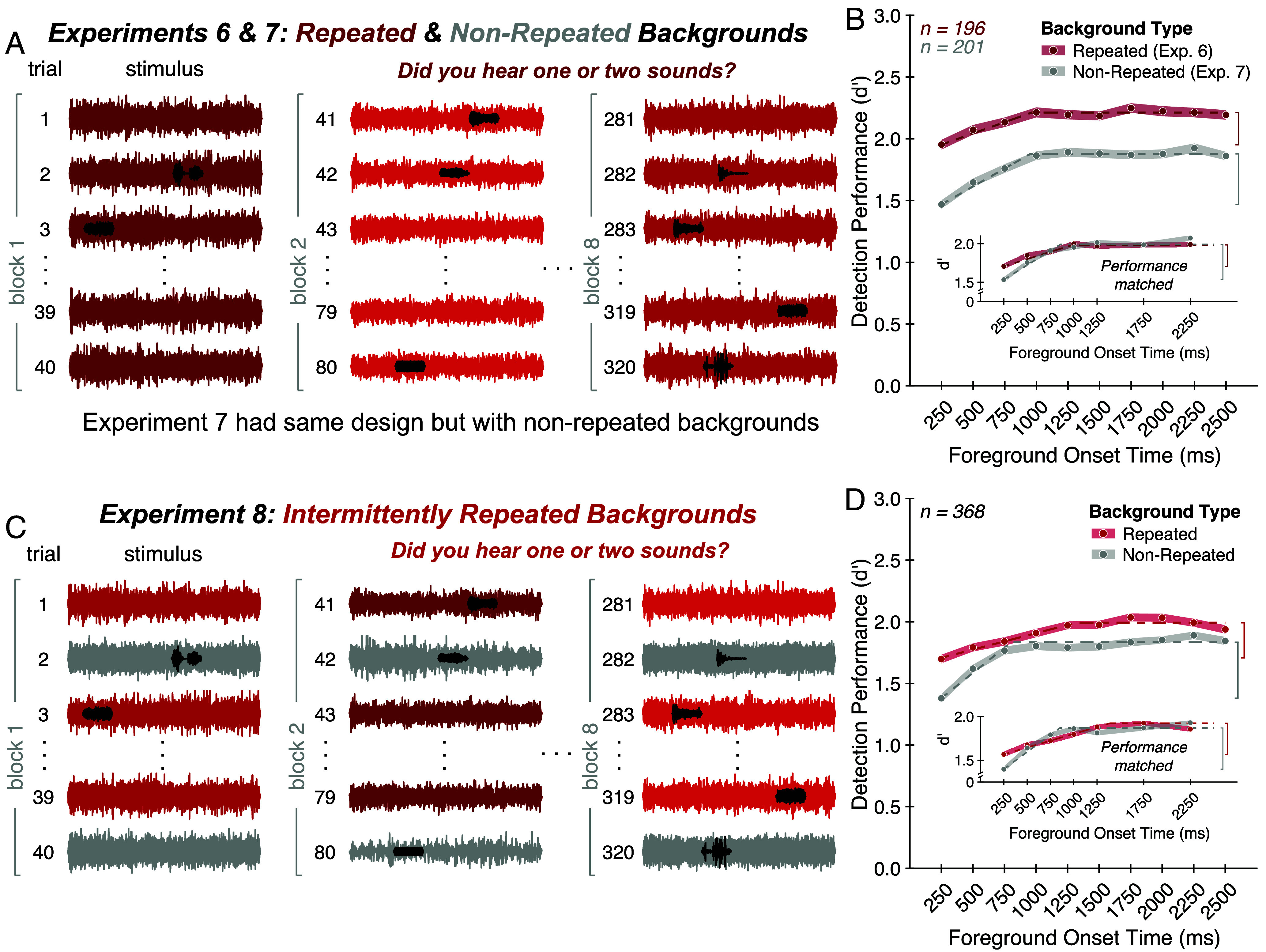
Experiments 6 to 8: Repetition of background noise enhances foreground detection. (*A*) Experiment 6 design. A background noise was repeated (red waveforms) on every trial in blocks of 40 trials, with each block containing a different repeating background noise (denoted by different shades of red). Participants judged whether the stimulus contained one or two sound sources. To ensure listeners would not benefit from learning the structure of the foregrounds, each foreground occurred only once with foregrounds and backgrounds paired randomly across participants. This design necessitated a companion experiment with similarly uncontrolled foreground–background pairings in which the backgrounds were not repeated across trials (Experiment 7; not shown). (*B*) Experiment 6 and 7 results. Average foreground detection performance (quantified as d′) is plotted as a function of foreground onset time for repeated (red circles) versus nonrepeated (gray circles) backgrounds. Shaded regions plot SE. Dashed lines plot elbow function fit. Vertical brackets denote the delay benefit. The *Inset* shows results after matching asymptotic performance across groups of participants. (*C*) Experiment 8 design. The experiment was identical to Experiment 6 except that background noises were repeated (red waveforms) on every other trial within a block with intervening trials containing nonrepeating backgrounds (gray waveforms). (*D*) Experiment 8 results. Same conventions as (*B*).

We first ran a variant of Experiment 1 in which a subset of the background noises (selected randomly for each participant) occurred repeatedly over the course of the experiment (Fig. 5*A*). A background noise was repeated on every trial in blocks of 40 trials, with each block containing a different repeating noise. We used unique noise exemplars for each repetition so that listeners would have to learn the statistical properties of the noise (28) rather than the specific exemplar (52, 53). We note that adaptation could, in principle, be expected to build up over the course of the block of repeated noise, potentially also accounting for altered performance. This experiment was thus not intended to distinguish noise schemas from adaptation, but rather to test a prediction of noise schemas in a simple setting before probing for benefits of repeated noises that might be less likely to be produced by adaptation (Experiment 8).

Each foreground sound occurred only once throughout the experiment to avoid the possibility that listeners might instead benefit from learning the structure of the foreground. As a result, we had to forego the controlled foreground–background pairings used in Experiments 1 to 5 and instead allowed foregrounds and backgrounds to be paired randomly across participants, while lowering the SNR to partially compensate for the decreased average spectral overlap between foreground and background. This design constraint necessitated a companion experiment (Experiment 7) with similarly uncontrolled foreground–background pairings in which the backgrounds varied across trials, with each of the 160 background noises occurring once with a foreground and once without a foreground, as in Experiment 1.

In the main analysis of interest, we found that performance was enhanced for repeating compared to nonrepeating background noises [Fig. 5*B*; main effect of background type: F(1,395) = 90.82, *P* < 0.001, $\eta_{\mathrm{partial}}^{2}=\;0.19$]. This enhancement developed over the course of a block in which the noises were repeated [*SI Appendix*, Fig. S3, main effect of first versus second half of trials within a block: F(1,195) = 17.44, *P* < 0.001, $\eta_{\mathrm{partial}}^{2}=\;0.08$]. Although an effect of foreground onset time remained evident when the noise was repeated [main effect of foreground onset time for Experiment 6: F(9,1755) = 10.75, *P* < 0.001, $\eta_{\mathrm{partial}}^{2}=\;0.05$], there was a significant interaction between foreground onset time and background repetition [F(9,3555) = 2.52, *P* = 0.01, $\eta_{\mathrm{partial}}^{2}=\;0.01$]. Specifically, the delay benefit was smaller for repeated backgrounds compared to nonrepeated backgrounds (significant difference in the delay benefit; *P* < 0.001 via permutation test, delay benefit difference: 0.15 in units of d′). To ensure the reduced delay benefit for repeating backgrounds was not driven by participants with near-ceiling performance, we ran a control analysis in which we selected groups of participants to have similar asymptotic performance using data from foreground onset times of 1,500, 2,000, and 2,500 ms (*Materials and Methods*), then measured the delay benefit using the data from the remaining foreground onset times for these participants. After matching asymptotic performance across groups of participants, the reduction in delay benefit persisted for repeated compared to nonrepeated backgrounds (Fig. 5 *B*, *Inset*, significant difference in delay benefit; *P* = 0.01 via permutation test, delay benefit difference: 0.17 in units of d′).

Overall, these results confirm one prediction of the schema-based account of noise robustness: Detection performance is improved for recurring backgrounds and less dependent on online noise estimation. We also note that these findings help reconcile the results in this paper with those of more traditional experimental paradigms, which repeat the same type of background noise throughout an experiment and find less pronounced temporal effects than those shown here.

We additionally note that the results seem to be qualitatively unaffected by whether the foreground–background pairings were controlled. The effect of foreground onset time was similar in Experiment 7 (uncontrolled pairings) compared to Experiment 1 (controlled pairings), with no significant interaction between the experiment and the effect of foreground onset time [*SI Appendix*, Fig. S4; F(9,2628) = 0.65, *P* = 0.75, $\eta_{\mathrm{partial}}^{2}=\;0.00$]. Both the timescale of improvement and the delay benefit were similar between the two experiments (no significant difference in elbow point: *P* = 0.72 via permutation test, elbow point difference: 41 ms; no significant difference in delay benefit: *P* = 0.50 via permutation test, delay benefit difference: 0.03 in units of d′).

### Experiment 8: Foreground Detection Is Enhanced for Intermittently Repeated Background Noises.

We next asked whether the benefit from recurring noises would be preserved across intervening stimuli, as might be expected if noise schemas are retained in memory, but not if the benefit reflects standard adaptation. In Experiment 8, the same type of background noise occurred on every other trial within a block (Experiment 8; Fig. 5*C*). As in Experiment 6, each block contained a different repeating background noise with unique noise exemplars for each repetition and each foreground sound was presented once with foregrounds and backgrounds paired randomly across participants.

We again found that performance was enhanced for repeating compared to nonrepeating background noises [Fig. 5*D*; main effect of background type: F(1,367) = 63.03, *P* < 0.001, $\eta_{\mathrm{partial}}^{2}=\;0.15$]. Additionally, there was again a significant interaction between the effect of foreground onset time and whether the background was repeated or not [F(9,3303) = 4.38, *P* < 0.001, $\eta_{\mathrm{partial}}^{2}=\;0.01$], such that the delay benefit was smaller for repeating compared to nonrepeating backgrounds (significant difference in delay benefit: *P* = 0.002 via permutation test, delay benefit difference: 0.17 in units of d′). This difference persisted after matching asymptotic performance across subsets of participants (Fig. 5 *D*, *Inset*, significant difference in delay benefit; *P* = 0.03 via permutation test, delay benefit difference: 0.13 in units of d′). These results suggest that noise schemas—representations of noise statistics that aid foreground detection—are built up over time, maintained across intervening stimuli, and lessen the dependence on online noise estimation.

### Experiment 9: Benefit of Background Exposure Is Reduced for Stationary Noise.

**Fig. 6.**
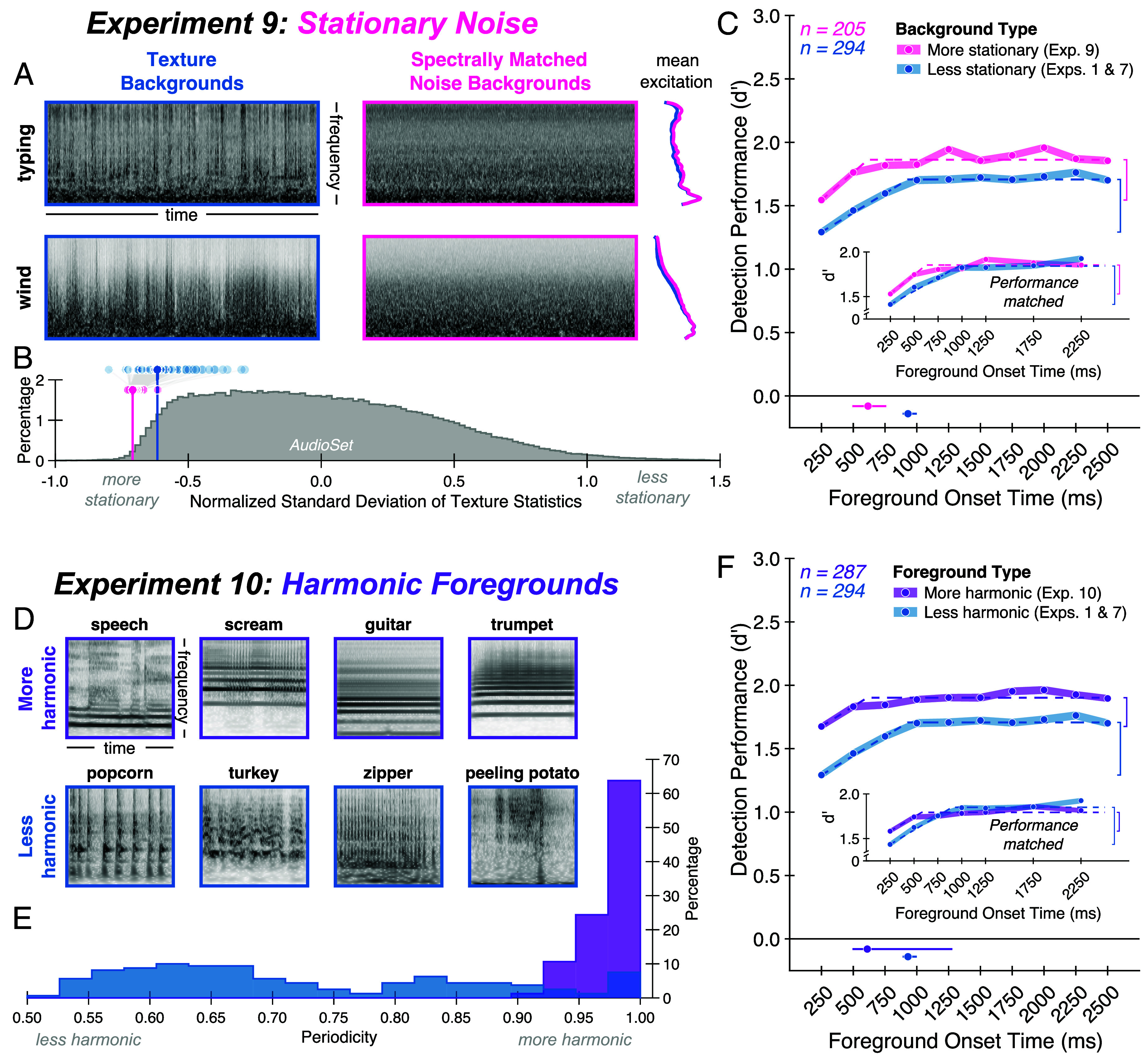
Experiments 9 and 10: Benefit of background exposure is reduced for stationary noise and harmonic foregrounds. (*A*) Example background noises from Experiment 9. The real-world texture backgrounds (*Left*, blue) used in Experiments 1 to 8 were replaced with spectrally matched noise (*Middle*, pink) to increase the noise stationarity. Backgrounds are displayed as cochleagrams (with darker gray indicating higher intensity) and mean excitation patterns (*Right*). (*B*) Stationarity of background noises. Shaded circles indicate a measure of stationarity (SD of texture statistics over time, normalized to account for increased variability of some statistics relative to others; *Materials and Methods*) for the texture backgrounds used in Experiments 1 to 8 (shown in blue) and the spectrally matched noise backgrounds used in Experiment 9 (shown in pink). Gray lines connect textures to their spectrally matched counterparts, illustrating that the spectrally matched noise is generally more stationary than its texture counterpart. Vertical lines indicate mean stationarity of background noises in each stimulus set. For comparison, a histogram of stationarity scores calculated from a large set of YouTube soundtracks (AudioSet; *Materials and Methods*) is shown in dark gray. Both sets of background noises are more stationary than the average soundtrack. (*C*) Experiment 9 results. Average foreground detection performance (quantified as d′) is plotted as a function of foreground onset time for more stationary (pink circles) versus less stationary (blue circles; obtained from pooled results of Experiments 1 and 7) backgrounds. Shaded regions plot SE. Dashed lines plot elbow function fit. Vertical brackets denote the delay benefit. Solid lines below the main axis plot one SD above and below the median elbow points, obtained by bootstrapping over participants; dots on these lines plot the fitted elbow points from the complete participant samples. The *Inset* shows results after matching asymptotic performance across groups of participants. (*D*) Example foreground sounds from Experiment 10. The foreground sounds used in Experiments 1 to 9 (*Bottom*, blue) were replaced with human vocalizations and musical instrument sounds (*Top*, purple). Foregrounds are displayed as cochleagrams. (*E*) Harmonicity of foregrounds. Harmonicity was quantified with a measure of waveform periodicity (*Materials and Methods*). Histograms of periodicity are shown for the set of human vocalizations and instrument sounds used in Experiment 10 (purple) and for the set of foregrounds used in Experiments 1 to 9 (blue). (*F*) Experiment 10 results. Average foreground detection performance (quantified as d′) is plotted as a function of foreground onset time for more harmonic (purple circles) versus less harmonic (blue circles; obtained from pooled results of Experiments 1 and 7) foregrounds. Conventions same as (*C*).

Across multiple tasks, we consistently found an improvement in performance over the initial second of exposure to the background. What factors might govern the timescale of this effect? A model that estimates statistics within an integration window (as in the model of Fig. 3) should exhibit improved performance up to the window width. One intuitive possibility is that the window width reflects a tradeoff between obtaining a good estimate of the background noise statistics (better for longer windows) and being able to resolve changes in these statistics (better for shorter windows). However, the accuracy with which statistics can be estimated for a given window size depends on the stability of the noise statistics over time (i.e., the stationarity of the noise). This observation raises the possibility that the optimal estimation window could be shorter for more stationary noise. To test whether these considerations might influence hearing in noise, we modified the stimuli from Experiment 1, replacing the real-world texture backgrounds with spectrally matched noises (Fig. 6*A*) to create noise backgrounds with increased stationarity. We quantified stationarity with a measure of the SD of texture statistics across time windows (17, 33, 34, 48) (*Materials and Methods*) and confirmed the increase in stationarity for spectrally matched noise backgrounds (Fig. 6*B*). Because the detection task is easier with more stationary noise, we reduced the SNRs of the foreground relative to the background to avoid ceiling performance.

As in our previous experiments, foreground detection performance improved with exposure to the background [Fig. 6*C*; main effect of foreground onset time: F(9,1836) = 25.99, *P* < 0.001, $\eta_{\mathrm{partial}}^{2}=\;0.11$]. However, the effect was more modest than that observed with more naturalistic noise [significant interaction between foreground onset time and stationarity: F(9,3636) = 2.69, *P* = 0.004, $\eta_{\mathrm{partial}}^{2}=\;0.01$]. In particular, the timescale of improvement was shorter for stationary noise backgrounds compared to texture backgrounds (significant difference in elbow point for Experiment 9 compared to pooled results from Experiments 1 and 7: *P* = 0.01 via permutation test, elbow point difference: 319 ms), and the delay benefit was reduced (significant difference in delay benefit: *P* = 0.01 via permutation test, delay benefit difference: 0.10 in units of d′). This reduction in delay benefit remained after matching asymptotic performance across groups of participants (Fig. 6 *C*, *Inset*, significant difference in delay benefit; *P* = 0.03 via permutation test, delay benefit difference: 0.11 in units of d′). These findings help to further reconcile the results of this paper with prior work that has predominantly used highly stationary synthetic noise and has found weaker effects of onset time. The temporal effects we nonetheless observed could reflect the fact that the background noise spectrum varied from trial to trial in our experiments (unlike most experiments in prior work).

As with other effects of stationarity on integration timescales (33), there are at least two computational accounts of these results. One is that there is a single statistical estimation window that changes in temporal extent depending on the input stationarity. Another is that there are multiple estimation windows operating concurrently (potentially estimating different statistical properties), with a decision stage that selects a window (or combination of windows) on which to base responses. For instance, by selecting the shortest window that enables a confident decision, a decision stage might determine that a shorter estimation window is most appropriate for stationary noise.

### Effect of Background Exposure Depends on Foreground–Background Similarity.

The possibility of concurrent estimation windows of different extents raised the question of whether the similarity of the foreground to the background could also influence the timescale of the effect of background exposure. Specifically, foregrounds that differ more noticeably from a background might be detectable with a less accurate model of the background that could be estimated with fewer samples (via a shorter estimation window). As one test of this possibility, we reanalyzed the results of Experiment 7, dividing trials into two groups whose foreground–background pairings differed in spectrotemporal similarity. We found a significant interaction between foreground onset time and spectrotemporal similarity [*SI Appendix*, Fig. S5; F(9,1791) = 3.90, *P* < 0.001, $\eta_{\mathrm{partial}}^{2}=\;0.02$], whereby the timescale of the noise exposure benefit was shorter for foreground–background pairs with lower spectrotemporal similarity (significant difference in elbow point: *P* = 0.003 via permutation test, elbow point difference: 526 ms). This result is consistent with the idea that there are multiple concurrent windows for estimating noise statistics, with shorter windows being used when they are sufficient for a decision. The result also provides further evidence that the estimation of noise properties aids the detection of the foreground. In particular, the result helps rule out the possibility that the effect of onset time reflects interference between the processing of the background and the detection of the foreground (as could in principle happen if the noise onset initiated some involuntary process that was not used to detect the foreground and that instead initially impaired processing of concurrent sounds).

### Experiment 10: Benefit of Background Exposure Is Reduced for Harmonic Foregrounds.

Motivated by the effect of foreground–background similarity shown in the preceding section, in the final experiment, we tested the effect of background exposure on the detection of approximately harmonic foregrounds. We avoided human vocalizations and music instrument sounds in our initial experiments on the grounds that they are more detectable in noise (5) compared to other sounds and so could have introduced another source of variation in detection performance. However, the analysis of foreground–background similarity suggested that harmonic sounds might produce weaker effects of background exposure; given their prevalence in both everyday life and prior auditory experiments, this seemed important to test. The experiment was identical to Experiment 7 except that the foreground sounds used in previous experiments were replaced with excerpts from human vocalizations and musical instrument sounds (Fig. 6*D*). We confirmed that these sounds were more harmonic than those used in the previous experiments, using a measure of waveform periodicity (54) (Fig. 6*E*; *Materials and Methods*).

A benefit of background exposure was evident for these (approximately) harmonic sounds [Fig. 6*F*; main effect of foreground onset time: F(9,2574) = 13.66, *P* < 0.001, $\eta_{\mathrm{partial}}^{2}=\;0.05$], but it was weaker than that observed for less harmonic sounds [significant interaction between foreground onset time and harmonicity: F(9,4374) = 4.25, *P* < 0.001, $\eta_{\mathrm{partial}}^{2}=\;0.01$]. Compared to the pooled results from Experiments 1 and 7, the elbow point was earlier (significant difference in elbow point: *P* = 0.03 via permutation test, elbow point difference: 317 ms) and the delay benefit was smaller (significant difference in delay benefit: *P* < 0.001 via permutation test, delay benefit difference: 0.19 in units of d′). This difference persisted after matching performance across experiments (Fig. 6 *F*, *Inset*; significant difference in delay benefit: *P* < 0.001 via permutation test, delay benefit difference: 0.20 in units of d′). The results again help to reconcile our findings with previous work using speech or tones that have found smaller effects, while also showing that the qualitative effects of background exposure remain evident with harmonic foregrounds.

## Discussion

We investigated whether internal models of environmental noise are used by the auditory system to aid the perception of natural foreground sounds in background noise. We found that the ability to detect, recognize, and localize foreground sounds in noise improved over the initial second of exposure to the background. This benefit of background exposure persisted even when participants knew the foreground sound they had to listen for. The benefit of prior noise exposure was robust to temporary changes in the background and was enhanced for recurring backgrounds, suggesting that noise schemas are built up and maintained over time. We found that an observer model designed to capture the statistics of ongoing background noise could account for the pattern of human behavioral performance observed in the foreground detection task. Finally, we found evidence for a window of noise estimation that varies depending on the stimulus characteristics, appearing shorter both for more stationary noise and when foreground sounds are sufficiently distinct (e.g., by virtue of being harmonic) from the background so as to not require a detailed model of the background properties. Overall, the results suggest that the auditory system leverages internal models of noise properties—noise schemas—to facilitate the estimation of other concurrent sounds and support noise-robust hearing.

### Relation to Prior Work.

We sought to distinguish between three candidate explanations for noise-robust hearing: fixed noise suppression, adaptive noise suppression, and internal modeling of noise schemas. Two lines of evidence have previously been presented in support of adaptive suppression (and contra fixed suppression). The first involves behavioral improvements in hearing abilities following exposure to a noise source (see ref. 38 for a review), with improvements occurring over approximately 500 ms. A second body of research involves evidence of neural adaptation to noise (13, 14, 16, 18, 23), with related modeling work suggesting that these adaptive responses could be explained by a mechanism that dynamically suppresses noise (23).

We built on this prior work in three respects. First, we found that foreground detection performance was robust to interruptions in the background and was enhanced for frequently recurring backgrounds. These findings are inconsistent with conventional adaptation to ongoing noise and instead suggest that noise properties are estimated and maintained over time. Second, we demonstrated that the benefits of noise exposure on behavior generalize to natural stimuli and everyday listening contexts. In these conditions, behavioral performance improved over a period roughly twice as long as previously reported, with performance plateauing around 1 s. These large effects appear to partly reflect the use of stimuli that vary across trials, realistic sources of noise, and diverse foreground sounds. We found smaller effects when noises repeated within an experiment (Experiments 6 and 8), when noise was more stationary (Experiment 9), and when foregrounds were harmonic (Experiment 10). These results reconcile our findings with previous work, which has tended to use a single type of highly stationary synthetic noise and harmonic foreground sounds, and which has seen smaller effects of time. Our results highlight the utility of assessing perception using natural stimuli, as it can reveal effects not fully evident with simpler traditional stimuli (55–57). Third, our experiments show that the temporal effects of exposure to background noise occur across multiple auditory tasks: detection, recognition, and localization.

The temporal dynamics of human task performance in noise could be explained by an observer model that estimates the statistics of ongoing background noise and detects foreground sounds as outliers from this distribution. This finding demonstrates that the observed improvement in task performance following noise exposure can result directly from a model of noise properties, and raises the question of how to reconcile our results with neurophysiological findings of noise suppression in the auditory system. We suggest that noise modeling and noise suppression are not mutually exclusive. One possibility is that the auditory system maintains parallel representations of sound: one in which noise properties are estimated and maintained and another in which noise is suppressed to yield a relatively invariant representation of the foreground. The first representation could be used to derive the second, such that as noise becomes more accurately estimated (e.g., with more exposure to the background), the foreground representation becomes enhanced, as we found here. The existence of such parallel representations is consistent with neurophysiological findings that noise stimuli are represented subcortically (58, 59) and preferentially drive neurons in the primary auditory cortex (60–62) but appear to be suppressed in the nonprimary auditory cortex (17, 63).

### Limitations.

The model presented here provides evidence that estimation of noise statistics could underlie aspects of hearing in noise, but in its present form is not a complete account of human perception in this setting. As noted earlier, we modeled noise with relatively simple distributions that do not completely capture the structure known to be present in real-world noise. Although the approach was sufficient for our purposes, more sophisticated models will be required to fully account for human performance. A complete model would also estimate the properties of any foreground sounds in addition to detecting their presence. Intuitively, one might adopt an “old plus new” (64) approach in which samples that deviate from the distribution of ongoing background noise are interpreted as a (“new”) foreground sound whose features can be estimated as the “residual” after accounting for the background noise properties. The model as implemented here also does not account for the enhanced foreground detection observed for interrupted or frequently recurring backgrounds (Experiments 5 to 8). However, some of these effects could potentially be modeled by incorporating a prior over noise properties that is continually updated over the course of the experiment.

### The Role of Texture in Auditory Scene Analysis.

Using real-world noise signals, we found that the ability to hear in noise improves over the initial second of exposure to the background noise—substantially longer than the timescale previously reported for analogous tasks with simpler experimental stimuli. This relatively long timescale is broadly consistent with the growing literature on sound texture perception. Sound textures are thought to be perceptually represented in the form of time-averaged summary statistics (28) computed using averaging mechanisms with a temporal extent that depends on the texture stationarity (33) but is generally on the order of seconds. Moreover, the detection of changes in texture statistics documented in previous studies improves across a temporal scale similar to that observed in our work (29, 30). Other experiments have indicated that texture properties are estimated separately from other sound sources (33) and are filled in when masked by other sounds (34). Our results provide further evidence that sound texture plays a critical role in auditory scene analysis, as its estimation benefits the detection, recognition, and localization of other concurrent sounds. This growing literature supports the idea that background noise properties are actively estimated by the auditory system even in the presence of other sound sources.

### Reconsidering the Role of Noise.

Sound textures are ubiquitous in everyday listening, constituting the background noise of many real-world auditory scenes. Yet research on hearing in noise has devoted relatively little attention to the role of noise itself. We consider hearing in noise as a form of auditory scene analysis in which listeners must segregate concurrent foreground and background sources from one another. From this perspective, noise is another source to be estimated rather than suppressed (65). However, the segregation of multiple sound sources is only possible because of the statistical regularities of natural sounds. Previous work has shown that human listeners can quickly detect repeating patterns in the acoustic input and use this structure to facilitate source segregation in artificial auditory scenes (66–68). The present results complement these findings by showing that the predictable statistical structure of noise is used to aid source segregation in natural auditory scenes.

### Future Directions.

Reverberation is another element of sound often thought of as a distortion that the auditory system must suppress to improve hearing in acoustic environments characteristically encountered in daily life (15, 69–71). It is analogously possible that the estimation, rather than suppression, of reverberation might help to recover the underlying sound source (72, 73). Thus, robustness to reverberation may be aided by an internal model of the statistical regularities that characterize real-world reverberation (72). Schemas of room reverberation may also be built up through short-term exposure to room-specific reverberation (74–77).

The computational principles described here are equally relevant to other sensory modalities. For instance, the detection or recognition of an object amid a cluttered visual scene can be viewed as a visual analogue of our hearing-in-noise experiments. As in hearing, the ability to visually recognize objects is impaired by clutter—a widely studied phenomenon known as visual crowding (78). Given that visual textures are thought to be represented by summary statistics averaged over space (79, 80), visual object recognition might be expected to improve with the size of a background texture region as background properties should be better estimated across larger spatial extents. Some preliminary evidence supports this hypothesis. Several studies from the visual crowding literature demonstrate a release from crowding when additional distractors or flankers are added to a display (81, 82). However, these studies are limited to relatively simple displays, and it remains to be seen whether such effects may be observed in more naturalistic settings.

## Materials and Methods

Methods are described in full detail in *SI Appendix*, *SI Materials and Methods*. The full methods section includes descriptions of experimental participants and procedures, stimulus generation, data analysis, statistical tests, and power analyses. All participants provided informed consent, and the Massachusetts Institute of Technology Committee on the Use of Humans as Experimental Subjects approved all experiments.

## Supplementary Material

Appendix 01 (PDF)

## Data Availability

All code and data are available at https://github.com/mcdermottLab/NoiseSchemas (83).
