# Supplementary material for: Noise schemas aid hearing in noise
Source: Proc Natl Acad Sci U S A. 2024 Nov 15;121(47):e2408995121. doi: 10.1073/pnas.2408995121 (PMC11588100; doi:10.1073/pnas.2408995121)
Supplement: Supplementary file 1 — Appendix 01 (PDF) [file pnas.2408995121.sapp.pdf]

Supporting Information for
Noise schemas aid hearing in noise

Jarrod M. Hicks[1,2,3] & Josh H. McDermott[1,2,3,4]
[1] Department of Brain and Cognitive Sciences, Massachusetts Institute of Technology
[2] McGovern Institute, Massachusetts Institute of Technology
[3] Center for Brains Minds and Machines, Massachusetts Institute of Technology
[4] Speech and Hearing Bioscience and Technology, Harvard University

Correspondence should be addressed to J.M.H. and J.H.M
Email: jmhicks@mit.edu; jhm@mit.edu

This PDF file includes:

SI Materials & Methods
Figures S1 to S5
Tables S1 to S3
SI References

SI Materials & Methods

Stimulus selection

Foreground sounds: To select foreground sounds for our experimental stimuli, we began with a set of 447 2-second-long recordings of natural sounds used in previous experiments from our laboratory (1, 2). Because harmonicity can aid hearing in noise (3), we manually screened these recordings to remove any sounds containing music, speech, or human vocalizations (e.g., screams or grunts). Some approximately harmonic sounds nonetheless remained in the stimulus set (e.g., alarms, various beeping electronics, animal vocalizations, etc.); see Fig. 6E. Additionally, we removed texture-like sounds (e.g., hairdryer or crumpling paper) to help ensure that selected foregrounds would be distinct from the sound texture backgrounds they were superimposed on. This left us with a set of 167 foreground sounds. These foreground sounds were used for Experiments 1-9. Experiment 10 used a different set of foreground sounds chosen to be approximately harmonic (see "Experiment 10" below).

Background sounds: To select background sounds for our experimental stimuli, we screened a large set of audio examples (AudioSet (4)) for sound textures. Specifically, we first screened the "unbalanced train set" within AudioSet by excluding 1) any sound whose label indicated the presence of speech or music (e.g., "whispering", "song", etc.; see Table S1 for list of excluded labels), 2) any sound from the "sourceless" branch of the ontology, 3) any sound less than 10 s in length, and 4) any sound with greater than 1% of values equal to zero. This resulted in a set of 222,560 sounds (which were used for computing the normalization values used in the stationarity measure described below). We then computed a measure of stationarity developed in previous work from our lab (5–7) for each sound within this set and excluded any sound with a stationarity score above 0, leaving us with a large set of 142,922 AudioSet "textures." From these AudioSet "textures", we selected relatively stationary sounds by keeping sounds with stationarity scores between -0.75 and -0.67 (approximately the 87th and 94th percentiles of the sounds with scores below 0). Additionally, we sought to avoid periodic textures (e.g., rhythmic clapping or waves crashing) because foreground detectability within such textures greatly depends on the timing of the foreground relative to the period of the background texture. We measured the periodicity of each AudioSet texture as in previous work (7) by measuring the normalized auto-correlation of the envelope of the stimulus waveform and selecting the maximum peak between 125 ms and 500 ms (2–8 Hz). We kept only those sounds whose periodicity fell within 0.05 and 0.075 (approximately the 1st and 7th percentiles across all AudioSet textures). The intersection of the stationary and non-periodic textures yielded 1511 textures. We note that the stationarity analyses that were subsequently performed in this paper used a slightly different stationarity measure than the one used for the initial screening described above (the new measure was similar in spirit to the old one, but used a different form of normalization). As a result, the stationarity scores referenced above differ slightly from those reported in Fig. 6B.

The background noises used in our experiments were textures synthesized from statistics measured from the (recorded) AudioSet sound textures. There were two reasons for this choice. First, textures recorded in natural environments often contain distinctive acoustic events arising from other sources. For example, a recording of a stream might contain faintly audible bird calls. Such additional sources would create confusion in the experiments involving detection or recognition. Second, in several experiments (Experiments 3, 6 and 8) we needed to present multiple exemplars of the same texture, and for this purpose required more than 10 s of audio. For each of the 1511 textures, we created 9-second-long synthetic exemplars using a standard texture synthesis method (8). We found that the synthesis procedure converged (average SNR of all statistic classes was 20 dB or higher) for 1285 textures and selected the background noises

from this set. We drew 3.25 s excerpts from these 9-second-long synthesized textures (see Foreground-background pairings below) to use in Experiments 1, 2, 4, 5, 7 and 10.

Stationarity measure: To quantify the stationarity of a sound for the analysis of Experiment 9, we computed a measure based on the standard deviation of texture statistics (8) across successive time windows (5–7, 9), based on the idea that stationary sounds have temporally stable statistical properties. Specifically, we first computed a set of texture statistics (subband mean, envelope mean, envelope standard deviation, envelope skew, envelope correlations, modulation band power, C1 modulation correlations, and C2 modulation correlations) for successive segments of a signal (using excerpt lengths of 0.125, 0.25, 0.5, 1, and 2 s). To put each of the statistics on the same scale, we then z-scored each statistic using the mean and standard deviation of each statistic calculated across the set of screened full-length AudioSet sounds (see "Background sounds" section above). To quantify how much each statistic changed across excerpts, we computed the standard deviation of each z-scored statistic across all excerpts of the same length. Because some statistics are intrinsically more variable than others, we computed a normalized measure of the variability of each statistic by dividing the computed standard deviations (separately for each statistic and excerpt length) by the average (i.e., expected) standard deviation across all the screened AudioSet sounds. To obtain a single measure of stationarity, we then averaged these normalized standard deviations across statistics and excerpt lengths. However, because some statistics classes contain more statistics than others, we first averaged the normalized standard deviations across all statistics within each class before averaging across the statistics classes (effectively weighting each statistics class equally) and excerpt lengths. The result is a normalized measure of statistic variability where smaller (i.e., more negative) values indicate greater stationarity.

Foreground-background pairings: In our initial experiments, experimental stimuli were generated from pairs of foregrounds and backgrounds selected to have similar long-term spectra to avoid large differences in foreground detectability across different foreground-background pairings. To select these pairs, we first created cochleagrams for each possible foreground and background sound. Cochleagrams were generated from the envelopes of a set of 38 bandpass filters (plus one low-pass and one high-pass channel) at a sampling rate of 500 Hz with tuning modeled on the human ear (8). Next, for each 2-second-long foreground sound, we randomly selected 100 0.5s cochleagram segments (from the entire 2s sound) and computed the Mahalanobis distance (D) between each foreground cochleagram segment and every background cochleagram. Specifically, we calculated the Mahalanobis distance for each point in time of the foreground cochleagram using the background cochleagram as the reference distribution, then averaged these distances over time: $D = \frac{1}{T}\sum_{t=1}^{T}\sqrt{(F_t - m)^T S^{-1}(F_t - m)}$, where F_t is one column of the foreground cochleagram at time t, m is the excitation pattern (time-averaged cochleagram) of the background, and S is the covariance of the background cochleagram. The Mahalanobis distance quantifies the difference between the foreground and background excitation patterns while accounting for the covariance structure among cochlear channels measured from the background. For every possible foreground-background pair, we stored the foreground segment with minimum Mahalanobis distance then used the Hungarian algorithm (10)) to pair each of the 167 foregrounds with a background sound such that the Mahalanobis distance across the pairings was minimized. Finally, we manually listened to each of the selected background textures and selected 3.25 s excerpts that subjectively sounded fairly uniform. We then selected 7 of these pairings to use as practice trials, leaving the remaining 160 foreground-background pairings to be used as experimental stimuli. Table S2 lists each of the foreground-background pairings; the sound waveforms for each foreground and background are provided in the data and code repository for this paper.

Experimental procedure for online participants
The condition-rich design of our experiments (e.g., 20 experimental conditions in Experiment 1), resulted in obtaining relatively few trials per condition per subject. To obtain the large sample sizes necessary to attain reliable results, we conducted our experiments (with the exception of Experiment 3) online using the Amazon Mechanical Turk and Prolific crowdsourcing platforms. Experiments 1 and 4 were conducted in 2021-2022 on Amazon Mechanical Turk. Experiments 2 and 5-10 were conducted in 2023-2024 on Prolific. Across multiple studies from our laboratory, we have found that online data can be of comparable quality to data collected in the lab provided a few modest steps are taken to standardize sound presentation, encourage compliance and promote task engagement (7, 11–15).

All participants provided informed consent and the Massachusetts Institute of Technology Committee on the Use of Humans as Experimental Subjects (COUHES) approved all experiments. Amazon Mechanical Turk participants were required to be in the United States, to have a HIT approval rate of greater than 95%, and to have had more than 100 HITs approved. Prolific participants were required to be in Canada, the United Kingdom, or the United States, to have an approval rate of greater than 95%, and to be fluent in English.

Participants were asked to perform the experiment in a quiet location and minimize external sounds as much as possible. Next, participants were instructed to set the computer volume to a comfortable level while listening to a calibration noise signal set to the maximum sound level presented during the main experimental task. Each experiment then began with a "headphone check" task to ensure participants were wearing headphones (16). Ensuring headphone use provides more standardized sound presentation across participants and helps to improve overall listening conditions by reducing external background noise. Following the headphone check task, each participant performed a set of practice trials for which feedback was provided after each response. The practice trials helped to ensure participants understood the task instructions and could perform the task correctly. In the main experiment, we incentivized good performance and task engagement by providing feedback after each trial and rewarding participants with a small bonus payment for each correct trial (17).

Exclusion criteria
During analysis, we screened out participants who were not able to perform the task by excluding those whose performance, averaged across conditions, was below a level that we expected every attentive and normal-hearing participant to achieve, provided they understood the instructions. Because the purpose of the experiments was to assess differences between conditions, rather than absolute performance, this exclusion procedure is neutral with respect to the hypotheses. For Experiments 1 and 4, participants were excluded if average detection performance (d') was below 0.6. For Experiment 2, participants were excluded if average recognition performance was below 40% correct. For Experiment 3, we planned to exclude participants whose average localization error was above 30° (as it turned out, all had error levels below this criterion). For Experiments 5-10, participants were excluded if average detection performance (d') was below 0.8. This exclusion criterion excluded between 0% and 17% of participants, depending on the experiment.

Experiment 1 (Detection)
<u>Stimuli</u>: For each of the 160 foreground-background pairs, we constructed stimuli in which the foreground appeared at each of 10 possible temporal positions (foreground onset times of 250, 500, 750, 1000, 1250, 1500, 1750, 2000, 2250, and 2500 ms) and each of 2 possible SNRs (-2

and -6 dB). This yielded a total of 3200 stimuli containing a foreground sound. We also created an additional 160 stimuli that consisted of the background noise only.

Procedure: The experiment consisted of 320 trials. Half of these trials included the 160 background noises without a foreground sound. The other half of these trials included each of the 160 foreground-background pairings randomly assigned to one of the 20 experimental conditions (10 foreground positions crossed with 2 SNRs). On each trial, participants judged whether the stimulus contained one or two sound sources.

Participants: A total of 200 participants were recruited through Amazon Mechanical Turk. Of these, 88 participants were excluded either because they failed the headphone check task, had self-reported hearing loss, withdrew from the experiment, or completed less than 90% of experimental trials. Finally, 19 participants were excluded due to low task performance (average d' < 0.6). This resulted in a total of 93 participants included in data analyses. Of these participants, 41 identified as female, 45 as male, and 1 as nonbinary (6 participants did not provide a response). The average age of participants was 37.3 (s.d. = 11.0). All participants were unique to this experiment.

Sample size: To determine the sample size necessary to yield stable results, we ran a pilot version of the experiment with 92 participants and calculated the split-half reliability of the average foreground detection performance as we varied the number of participants included in the analysis. The pilot experiment was identical to the actual experiment apart from having 3s background noises (rather than 3.25s as in the actual experiment). Split-half reliability was computed by randomly splitting the sample in half, measuring the Pearson correlation between average performance results in each half, and then applying the Spearman-Brown correction. Because we were primarily interested in the effect of foreground onset time, we measured the split-half reliability separately for each SNR condition then averaged the split-half reliabilities across SNR conditions. Additionally, since the estimated reliability depends on the random split of participants, we repeated this procedure for 10,000 random splits. Because the resulting distribution of reliabilities was skewed, we applied the Fisher z-transform to make the distribution approximately normal. We then took the mean of the Fisher z-transformed distribution (i.e., mean across all random splits) and applied the inverse Fisher z-transformation to obtain our final measure of split-half reliability. We performed this procedure as we varied the number of included participants and found that split-half reliability increased from 0.40 with 10 participants to 0.89 with 92 participants. We fit a curve to these reliabilities and extrapolated that a sample size of 94 participants would be needed to achieve a split-half reliability of at least 0.9. We targeted this sample size, but due to the nature of the screening procedure and the need to collect online data in batches of participants, the actual sample was slightly below this target.

Statistics and data analysis: We calculated a hit rate for each of the 20 experimental conditions (10 foreground onset times crossed with 2 SNRs) and a single false alarm rate using the background-only trials. Detection performance was quantified as d': $d' = \Phi^{-1}(\text{Hit Rate}) - \Phi^{-1}(\text{False Alarm Rate})$ where Φ^{-1} is the inverse CDF of the standard normal distribution. We performed a repeated measures analysis of variance (ANOVA) to analyze the effect of foreground onset time and SNR on foreground detection performance. We assumed data was normally distributed and evaluated this by eye. Mauchly's test indicated that the assumption of sphericity had not been violated. For each main effect and interaction of interest, we reported F-statistics, p-values and $\eta^2_{partial}$. To quantitatively estimate the timescale of improvement with exposure to the background, we fit an elbow function to the results averaged over SNRs. The elbow function was a piecewise linear function consisting of a rise and plateau: $f(t) = \begin{cases} at + b, & t < c \\ ac + b, & t \geq c \end{cases}$, where a

is the slope of the rise, b is the intercept of the rise, c is the transition from rise to plateau (i.e., the "elbow point") and t is time. We fit the elbow function by minimizing the absolute error between the estimated elbow function and the data. To obtain a confidence interval around the location of the elbow point, we bootstrapped over participants 10,000 times.

Experiment 2 (Recognition)
Stimuli: We used the same stimuli as in Experiment 1, including only those that contained a foreground sound.

Procedure: Each participant heard one trial for each of the 160 foreground-background pairings randomly assigned to one of the 20 experimental conditions (10 foreground positions crossed with 2 SNRs). Thus, the experiment consisted of 160 trials. On each trial, participants were asked to identify the foreground by selecting a text label from five options. One option was the correct label of the foreground, and the remaining options were chosen randomly from the labels of the other foreground sounds in the stimulus set.

Participants: A total of 409 participants were recruited through Prolific. Of these, 133 participants were excluded either because they failed the headphone check task, had self-reported hearing loss, withdrew from the experiment, or completed less than 90% of experimental trials. Finally, 15 participants were excluded due to low task performance (average recognition performance < 40% correct). This resulted in a total of 261 participants included in data analyses. Of these participants, 123 identified as female, 134 as male, and 2 as nonbinary (2 participants did not provide a response). The average age of participants was 38.8 (s.d. = 12.1). All participants were unique to this experiment.

Sample size: To determine the sample size necessary to yield stable results, we ran a pilot version of the experiment with 103 participants and calculated the split-half reliability of the average foreground recognition performance as we varied the number of participants included in the analysis. The pilot experiment was identical to the actual experiment apart from having 3s background noises (rather than 3.25s as in the actual experiment). The procedure for determining sample size was identical to that of Experiment 1. We found that split-half reliability increased from 0.07 with 10 participants to 0.53 with 102 participants. We fit a curve to these reliabilities and extrapolated that a sample size of 252 participants would be needed to achieve a split-half reliability of at least 0.9. We targeted this sample size, but due to the nature of the screening procedure, the actual sample was slightly above the target sample size.

Statistics and data analysis: We performed a repeated measures ANOVA to analyze the effect of foreground onset time and SNR on foreground recognition performance (quantified as precent correct). We assumed data was normally distributed and evaluated this by eye. Mauchly's test indicated that the assumption of sphericity had not been violated. For each main effect and interaction of interest, we reported F-statistics, p-values and $\eta^2_{partial}$. The procedure for fitting the elbow function was identical to that of Experiment 1.

Experiment 3 (Localization)
Stimuli: For each of the 160 background noises, we synthesized five unique 7-second-long exemplars and cut each exemplar into two 3.25-second-long sounds to yield a total of 10 unique waveforms for each background noise. We chose to synthesize 7-s exemplars rather than the 9-s exemplars used to generate stimuli in Experiments 1 and 2 because it reduced the time for synthesis while still enabling two excerpts to be cut from each exemplar. On a given trial, these 10 noise exemplars were played from 10 randomly chosen speakers to create diffuse background

noise. Each background noise was played at a level 52 dBA such that the total level of background noise was 62 dBA. The foreground sounds were identical to the 0.5s clips used in previous experiments and were played at a random speaker location (distinct from the 10 locations of the background noise) at a level of 50 dBA (i.e., at an SNR of -12 dB).

Procedure: Each participant heard one trial for each of the 160 foreground-background pairings, randomly assigned to one of the five experimental conditions (foreground onset times of 250, 750, 1250, 1750, and 2250 ms). Participants were instructed to fixate on the speaker directly in front of them, with their head still, for the duration of sound presentation. At the end of the sound presentation, participants could move their head to note the label of the speaker from which they judged the foreground sound to have played from. This label was entered using a keyboard. Participants were then instructed to reorient to the speaker directly in front of them before beginning the next trial. Trials were presented in two blocks of 80 trials with a short break between the blocks.

Participants: A total of 22 participants were recruited from the area around Cambridge, MA. Of these participants, 7 identified as female and 15 as male. The average age of participants was 26.4 (s.d. = 3.6). All participants were unique to this experiment. All participants provided informed consent and the Massachusetts Institute of Technology Committee on the Use of Humans as Experimental Subjects (COUHES) approved this experiment. No participants were excluded due to low task performance (average localization error > 30°).

Sample size: To determine an appropriate sample size, we performed a power analysis using G*Power (18). We sought to be 90% likely to detect an effect as big as that observed in Experiment 1, at a $p<0.01$ significance level using a repeated measures ANOVA with 5 repeated measurements (foreground onset times), assuming sphericity and a correlation among repeated measures of 0.2 (estimated from Experiment 1). This yielded a target sample size of 17 participants. We ran somewhat more than this to be conservative.

Statistics and data analysis: We performed a repeated measures ANOVA to analyze the effect of foreground onset time on foreground localization performance. Localization performance was quantified as the absolute localization error in azimuth. We assumed data was normally distributed and evaluated this by eye. Mauchly's test indicated that the assumption of sphericity had not been violated. For the main effect of interest, we reported F-statistics, p-values and $\eta^2_{partial}$. The procedure for fitting the elbow function was identical to that of Experiment 1.

Experiment 4 (Cued Detection)

Stimuli: The stimuli were identical to those of Experiment 1 but with lower SNRs (-5 and -8 dB). The cue sound was always the same waveform as the foreground sound that could appear within the background, the only difference being that the foreground amplitude was scaled to achieve the desired SNR for that trial. The cue was presented at the same level as the background, and thus differed in level from the foreground.

Procedure: The experiment consisted of 320 trials. On each trial, participants first heard a foreground sound in isolation (the "cued sound"), followed by continuous background noise. Half of the trials contained the cued foreground sound superimposed somewhere on the background noise, randomly assigned to one of the 20 experimental conditions (10 foreground positions crossed with 2 SNRs). Participants judged whether the stimulus contained the cued sound.

Participants: A total of 240 participants were recruited through Amazon Mechanical Turk. Of these, 81 participants were excluded either because they failed the headphone check task, had

self-reported hearing loss, withdrew from the experiment, or completed less than 90% of experimental trials. Finally, 23 participants were excluded due to low task performance (average d' < 0.6). This resulted in a total of 136 participants included in data analyses. Of these participants, 61 identified as female, 68 as male, and 1 as nonbinary (6 participants did not provide a response). The average age of participants was 38.5 (s.d. = 11.3). All participants were unique to this experiment.

<u>Sample size</u>: To determine the sample size necessary to yield stable results, we ran a pilot version of the experiment with 95 participants and calculated the split-half reliability of the average foreground detection performance as we varied the number of participants included in the analysis. The pilot experiment was identical to the actual experiment apart from having 3s background noises (rather than 3.25s as in the actual experiment) and SNRs of -2 and -6 dB (rather than -5 and -8 dB in the actual experiment). The procedure for determining sample size was identical to that of Experiment 1. We found that split-half reliability increased from 0.37 with 10 participants to 0.88 with 94 participants. We fit a curve to these reliabilities and extrapolated that a sample size of 105 participants would be needed to achieve a split-half reliability of at least 0.9. We targeted this sample size, but due to the nature of the screening procedure and the need to collect online data in batches of participants, the actual sample was slightly above the target sample size.

<u>Statistics and data analysis</u>: We calculated a hit rate for each of the 20 experimental conditions (10 foreground onset times crossed with 2 SNRs) and a single false alarm rate using the background-only trials, then quantified detection performance as d'. We performed a repeated measures ANOVA to analyze the effect of foreground onset time and SNR on foreground detection performance. We assumed data was normally distributed and evaluated this by eye. Mauchly's test indicated that the assumption of sphericity had not been violated. For each main effect and interaction of interest, we reported F-statistics, p-values and $\eta^2_{partial}$. The procedure for fitting the elbow function was identical to that of Experiment 1.

Observer model

<u>Overview</u>: First, an input sound waveform is passed through a standard model of auditory processing consisting of two stages: a peripheral stage modeled after the cochlea, yielding a "cochleagram", followed by a set of spectrotemporal filters (inspired by the auditory cortex) that operate on the cochleagram, yielding time-varying activations of different spectrotemporal features. Next, a probability distribution is estimated from the filter activations over a past time window. This distribution is then used to evaluate the surprisal of samples in a present time window. The process is then stepped forward in time and repeated, resulting in a set of surprisal values for each time point of the stimulus. Finally, this surprisal curve is compared to a time-varying decision threshold to decide whether a foreground sound is present.

<u>Cochleagram</u>: Cochleagrams were computed with a set of 40 filters (38 bandpass filters plus one low-pass and one high-pass filter). Filter cutoffs were evenly spaced on an ERB-scale (19) and thus mirrored the frequency resolution believed to characterize the human cochlea. Filters had transfer functions that were a half-cycle of a cosine function. The cochleagram resulted from the following sequence of steps (8). First, the filters were applied to the audio signal (at an audio sampling rate of 20000 Hz), yielding subbands. Second, subband envelopes were computed using the Hilbert transform. Third, the subband envelopes were passed through a compressive nonlinearity (by raising them to a power of 0.3). Fourth, the compressed envelopes were downsampled to a sampling rate of 2000 Hz.

Spectrotemporal filters: We selected spectrotemporal filters that were principal components of a large set of natural textures, as these captured the variance within natural background sounds. We first extracted 100 random 50-ms-long segments from the cochleagram representation of 1000 sound textures not used in our experiments, and then ran principal component analysis on these cochleagram segments. We found that 541 principal components were sufficient to explain 95% of the variance in the random segments and subsequently used these components as the spectrotemporal filters for our model. The filter activations were the dot product of the filter with the stimulus cochleagram.

Surprisal: Surprisal is defined as the negative log-probability of an event. Because we model filter activations using a continuous, normal distribution, we calculate surprisal using the negative log-density. For a univariate normal random variable $X \sim \mathcal{N}(\mu, \sigma^2)$, surprisal can be written as:

$$S(x) = -\ln\big(p(x)\big) = \frac{1}{2}\left(\frac{x-\mu}{\sigma}\right)^2 + \ln(\sigma) + \ln\big(\sqrt{2\pi}\big) = \frac{1}{2}(D)^2 + \ln(\sigma) + \ln\big(\sqrt{2\pi}\big),$$

where D is the Mahalanobis distance. Thus, any event that occurs with low likelihood will have high surprisal. On the grounds that foreground sounds should be unlikely under a distribution of the background, our model detects the presence of a foreground sound by tracking when the surprisal exceeds some criterion threshold. However, because the surprisal scales with the natural logarithm of the standard deviation, any threshold used for this purpose must similarly scale with the standard deviation of the background. In practice, rather than scale the decision threshold for each stimulus, we instead scale the surprisal by subtracting off the standard deviation term then use a fixed decision threshold across all stimuli.

Distribution fitting procedure: Due to the large number of spectrotemporal filters used, fitting a single high-dimensional joint distribution to the activations of all filters was intractable. Thus, we assumed activations across filters to be independent and fit separate univariate distributions to each filter's activations. In particular, we assumed filter activations were univariate Gaussians. We estimated the mean and variance of the activations within a past window and used these values to calculate the surprisal in a present window (averaging the surprisal over each time point within the window). We repeated this procedure at a sequence of time points, stepping forward in increments of 10 ms. This yielded a surprisal curve (surprisal over time) for each spectrotemporal filter. We then averaged across filters to yield the final surprisal curve. The size of the past window over which distributional parameters are estimated is a model hyperparameter. We tested past window sizes of 500, 750, 1000, 1250, 1500, 1750, 2000, 2250 and 2500 ms and present window sizes of 100, 250 and 500 ms. We found the 1000 ms past window and 500 ms present window to give the best fit with human results, as measured by the correlation with human results. The latter value is intuitively sensible given the 500 ms foreground duration. Figure 3 shows results for these window lengths.

Boundary handling: Boundaries pose a challenge for the estimation process in our model (and for the human perceptual system), for two reasons. First, at the onset of a stimulus, there is not yet enough stimulus history with which to estimate distribution parameters for the computation of surprisal (because there are not enough data points to reliably estimate parameters). Second, the filter activations contain boundary artifacts caused by the stages of filtering applied to the stimulus onset. We mitigated these issues by taking a weighted average of the estimated distributional parameters ($\hat{\theta}_t$ at time t) and a prior (π_t at time t) whenever the available stimulus history is less than the past window size (l in samples) over which parameters are estimated. Because the model was fit to the activations of spectrotemporal filters derived from PCA, the prior on the mean was 0 and the prior on the variance was given by the variance of each principal component across the set of random texture segments from which the principal components were computed (see "Spectrotemporal filters" section above). The weight (w_t at time t) was linearly relaxed from full

weight on the prior at stimulus onset to full weight on the estimated parameters once the available stimulus history was equal to the size of the past window. Thus, the model parameters (θ_t at time t) were given by:

$$\theta_t = \hat{\theta}_t(1 - w_t) + \pi_t w_t$$

where $w_t = \begin{cases} 1 - \frac{n_t}{l} & \text{if } n_t < l \\ 0 & \text{otherwise} \end{cases}$ and n_t is the number of samples available at time t.

Time-varying decision threshold: To determine a decision threshold, we ran the model on 100 random 3.25s excerpts of the 160 textures used in our experiments (see "Simulation of Experiment 1" below). This yielded a total of 16,000 surprisal curves. Then, for each point in time, we took the mean and standard deviation across all surprisal curves to quantify the distribution of surprisal in the absence of a foreground sound. The main idea is that surprisal values greater than that expected by chance (i.e., falling in the tail of this distribution) should indicate the presence of a foreground sound. We thus took the mean plus some number of standard deviations as the decision threshold. The number of standard deviations was chosen via grid search (1000 samples linearly spaced between 0.5 and 5) to best match the model's false alarm rate to that of human participants in Experiment 1. This was done separately for each set of model hyperparameters (i.e., past and present window sizes).

Decision rule: For each point in time, we evaluated whether the measured surprisal exceeded the decision threshold. The model decided a foreground sound was present if the surprisal exceeded the decision threshold for at least 50% of the time in any 500ms window.

Simulation of Experiment 1 (Fig. 3B):
Because the model could be run on arbitrarily many stimuli, we opted to show the model results in the limit of a very large amount of data. We simulated the experiment on a larger set of stimuli obtained by generating multiple texture exemplars for each of the background textures used in the human experiments. The stimuli were otherwise identical to those used in Experiment 1. To generate these stimuli, we synthesized 10 unique exemplars of each background noise texture then randomly took 10 different excerpts from each to yield a total of 100 unique excerpts of each background noise. We then ran the model on each of the 3,360 possible stimulus configurations (see "Stimuli" section in Experiment 1 above) for all 100 excerpts of a given background to yield model responses to a total of 336,000 stimuli. To provide a sense of the variability in model results for different subsets of stimuli, we computed model performance (quantified as d') over 10,000 subsets of the total 336,000 stimuli. Specifically, for each of the 20 experimental conditions, one stimulus was chosen randomly for each of the 160 foreground-background pairings and a model hit rate was computed from these 160 trials. Thus, a total of 3,200 (20 conditions x 160 pairings) trials were used to calculate the model hit rates for each experimental condition. To compute a model false-alarm rate, we randomly selected 20 background-only stimuli for each of the 160 backgrounds, giving another 3,200 trials. Together, this yielded a total of 6,400 stimuli (with half containing a foreground) for which performance was evaluated at each bootstrapped sample. Final model performance was taken as the mean performance across the 10,000 bootstrapped samples.

Experiment 5a (Short Interruptions in Background Noise)
Stimuli: For each of the 160 foreground-background pairs, we constructed 4-second-long stimuli in which the middle 500 ms of background noise was replaced with either silence or white noise (12 dB higher in level relative to the background). The foreground sound appeared at each of 8 possible temporal positions (foreground onset times of 250, 500, 750, 1000, 2500, 2750, 3000, and 3250 ms), at an SNR of -2 dB. This yielded a total of 2560 stimuli containing a foreground

sound. We also created an additional 320 stimuli that consisted of the background noise only with each of the two possible "interrupters" (silence or white noise).

Procedure: The experiment consisted of 320 trials. Half of these trials presented the 160 background noises without a foreground sound, randomly assigned to one of the two interrupter conditions. The other half of these trials included each of the 160 foreground-background pairings randomly assigned to one of the 16 experimental conditions (8 foreground positions crossed with 2 interrupter types). Participants were instructed to ignore the interrupter and judge whether the stimulus contained one or two sound sources.

Participants: A total of 105 participants were recruited through Prolific. Of these, 27 participants were excluded either because they failed the headphone check task, had self-reported hearing loss, withdrew from the experiment, or completed less than 90% of experimental trials. No participants were excluded due to low task performance (average d' < 0.8). This resulted in a total of 78 participants included in data analyses. Of these participants, 45 identified as female, 32 as male, and 1 as nonbinary. The average age of participants was 38.3 (s.d. = 12.2). All participants were unique to this experiment.

Sample size: To determine the sample size necessary to yield stable results, we ran a pilot version of the experiment with 57 participants and calculated the split-half reliability of the average foreground detection performance for foreground onset times prior to the interrupter as we varied the number of participants included in the analysis. The pilot experiment was identical to the actual experiment apart from being run on Mechanical Turk rather than Prolific. At the time the pilot experiment was run, data quality on Mechanical Turk had declined due to an uptick in fraudulent workers, and so we opted to run the actual experiment on Prolific but still considered the Mechanical Turk data to be reasonable as a pilot. The procedure for determining sample size was identical to that of Experiment 1. We found that split-half reliability increased from 0.40 with 10 participants to 0.84 with 56 participants. We fit a curve to these reliabilities and extrapolated that a sample size of 78 participants would be needed to achieve a split-half reliability of at least 0.9.

Statistics and data analysis: We calculated a hit rate for each of the 16 experimental conditions (8 foreground onset times crossed with 2 interrupter types) and false alarm rates using the background-only trials for each interrupter type, then quantified detection performance as d'. We performed a repeated measures ANOVA to analyze the effect of interrupter type and foreground position (relative to the interrupter) on foreground detection performance. We assumed data was normally distributed and evaluated this by eye. Mauchly's test indicated that the assumption of sphericity had not been violated. For each main effect and interaction of interest, we reported F-statistics, p-values and $\eta^2_{partial}$.

Experiment 5b (Longer Interruptions in Background Noise)
Stimuli: The stimuli were created in a manner similar to that of Experiment 5a. Previous work using EEG to measure adaptation in auditory-evoked cortical potentials in humans found that the recovery from adaptation (in silence) followed an exponential function with a time-constant of around 1300 ms (20). Thus, it seemed possible that the timescale of recovery from adaption exceeded the duration of the 500 ms interrupter used in Experiment 5a, causing the benefit of background exposure to persist across the interruption. Thus, in Experiment 5b, we increased the duration of the interrupter to 1500 ms. For each of the 160 foreground-background pairs, we constructed 5-second-long stimuli in which the middle 1500 ms of background noise was replaced with either silence or white noise (12 dB higher in level relative to the background). Because it seemed plausible that gaps between the noise and the background texture might make the noise more salient, making for a stronger test, the first and last 125 ms of the white noise interrupter

was replaced with silence. The foreground sound appeared at each of 8 possible temporal positions (foreground onset times of 250, 500, 750, 1000, 3500, 3750, 4000, and 4250 ms), at an SNR of -2 dB. This yielded a total of 2560 stimuli containing a foreground sound. We also created an additional 320 stimuli that consisted of the background noise only with each of the two possible "interrupters" (silence or white noise).

Procedure: The procedure was identical to that of Experiment 5a.

Participants: A total of 121 participants were recruited through Prolific. Of these, 49 participants were excluded either because they failed the headphone check task, had self-reported hearing loss, withdrew from the experiment, or completed less than 90% of experimental trials. Finally, 1 participant was excluded due to low task performance (average d' < 0.8). This resulted in a total of 71 participants included in data analyses. Of these participants, 31 identified as female, 39 as male, and 1 as nonbinary. The average age of participants was 34.3 (s.d. = 10.2). All participants were unique to this experiment.

Sample size: Because we planned to compare the results of Experiment 5b to that of Experiment 5a, we targeted the size of the sample collected in Experiment 5a (n=78), but due to the nature of the screening procedure, the actual sample was slightly below the target sample size.

Statistics and data analysis: Like Experiment 5a, we calculated a hit rate for each of the 16 experimental conditions (8 foreground onset times crossed with 2 interrupter types) and false alarm rates using the background-only trials for each interrupter type, then quantified detection performance as d'. We performed a repeated measures ANOVA to analyze the effect of interrupter type and foreground position (relative to the interrupter) on foreground detection performance. We assumed data was normally distributed and evaluated this by eye. Mauchly's test indicated that the assumption of sphericity had not been violated. To compare foreground detection performance following different interrupter durations (between Experiments 5a and 5b), we also performed a mixed model ANOVA with interrupter type as a within-subject factor and interrupter duration as a between-subject factor, including only onset times after the interruption in each experiment. For each main effect and interaction of interest, we reported F-statistics, p-values and $\eta^2_{partial}$.

Experiment 6 (Repeating Background Noises on Every Trial)

Stimuli: For each of the 160 background noises, we synthesized 20 7-second-long exemplars and cut each exemplar into two 3.25-second-long sounds to yield a total of 40 unique waveforms for each background noise. For each of these 6,400 unique background noise waveforms, each of the 160 foregrounds could appear at each of 10 possible temporal positions (foreground onset times of 250, 500, 750, 1000, 1250, 1500, 1750, 2000, 2250, and 2500 ms) at an SNR of -8 dB, yielding a total of 10,240,000 possible stimuli containing a foreground sound and 6,400 possible stimuli consisting of background noise only. Rather than create all possible experimental stimuli, we pre-generated enough stimulus sets (see Procedure below) such that each participant in our sample would receive a unique set, generating only the stimuli needed for these sets.

Procedure: For each subject, we randomly selected 8 of the 160 possible backgrounds to repeat on every trial in blocks of 40 trials. On half of these trials, the background noise appeared in isolation. The other half of these trials also contained a randomly selected foreground randomly assigned to one of the foreground onset time conditions such that each foreground onset time condition occurred twice during a block. Each background noise was a unique exemplar, and foregrounds were never repeated. The order of the blocks was chosen at random, as was the

order of stimuli within a block. On each trial, participants judged whether the stimulus contained one or two sound sources and were not explicitly informed that backgrounds would repeat.

Participants: A total of 289 participants were recruited through Prolific. Of these, 93 participants were excluded either because they failed the headphone check task, had self-reported hearing loss, withdrew from the experiment, or completed less than 90% of experimental trials. No participants were excluded due to low task performance (average d' < 0.8). This resulted in a total of 196 participants included in data analyses. Of these participants, 90 identified as female, 100 as male, and 6 as nonbinary. The average age of participants was 36.5 (s.d. = 11.9). All participants were unique to this experiment.

Sample size: We targeted the same sample size as in Experiment 7 (which is presented later in the text, but which was in practice run first), but due to the nature of the screening procedure, the actual sample was slightly below the target sample size.

Statistics and data analysis: We calculated a hit rate for each of the 10 experimental conditions (10 foreground onset times) and a single false alarm rate using the background-only trials, then quantified detection performance as d'. We performed a repeated measures ANOVA to analyze the effect of foreground onset time on foreground detection performance. We assumed data was normally distributed and evaluated this by eye. Mauchly's test indicated that the assumption of sphericity had not been violated. For each main effect and interaction of interest, we reported F-statistics, p-values and $\eta^2_{partial}$.

Experiment 7 (Non-repeated Background Noises with Random Foreground Pairings)
Stimuli: For each of the 160 background noises from Experiment 1, we constructed stimuli in which each of the 160 foregrounds appeared at each of 10 possible temporal positions (foreground onset times of 250, 500, 750, 1000, 1250, 1500, 1750, 2000, 2250, and 2500 ms) at an SNR of -8 dB. This yielded a total of 256,000 stimuli containing a foreground sound. We also created an additional 160 stimuli that consisted of the background noise only.

Procedure: The experiment consisted of 320 trials. Half of these trials included the 160 background noises without a foreground sound. The other half of these trials contained a randomly selected foreground randomly assigned to one of the foreground onset time conditions. On each trial, participants judged whether the stimulus contained one or two sound sources.

Participants: A total of 361 participants were recruited through Prolific. Of these, 158 participants were excluded either because they failed the headphone check task, had self-reported hearing loss, withdrew from the experiment, or completed less than 90% of experimental trials. Finally, 2 participants were excluded due to low task performance (average d' < 0.8). This resulted in a total of 201 participants included in data analyses. Of these participants, 82 identified as female, 112 as male, and 5 as nonbinary (2 participants did not provide a response). The average age of participants was 37.1 (s.d. = 12.0). All participants were unique to this experiment.

Sample size: Because we expected that the randomized foreground-background pairings used in this experiment would increase the variability of the results, we targeted double the sample size of Experiment 1 (n=93) to help ensure sufficient power. Due to the nature of the screening procedure, the actual sample was slightly above the target sample size.

Statistics and data analysis: We calculated a hit rate for each of the 10 experimental conditions (10 foreground onset times) and a single false alarm rate using the background-only trials, then quantified detection performance as d'. We performed a repeated measures ANOVA to analyze

the effect of foreground onset time on foreground detection performance. We assumed data was normally distributed and evaluated this by eye. Mauchly's test indicated that the assumption of sphericity had not been violated. To compare foreground detection performance for repeated (Experiment 6) versus non-repeated (Experiment 7) backgrounds, we performed a mixed model ANOVA with foreground onset time as a within-subject factor and background type as a between-subject factor. To compare foreground detection performance for controlled (Experiment 1) versus non-controlled (Experiment 7) foreground-background pairings, we performed a mixed model ANOVA with foreground onset time as a within-subject factor and foreground-background pairing type as a between-subject factor. For each main effect and interaction of interest, we reported F-statistics, p-values and $\eta^2_{partial}$. To estimate the overall magnitude of improvement in detection performance with foreground onset time, we fit an elbow function to the results and quantified the delay benefit as the difference between the values of the elbow function at the first (250 ms) and last (2500 ms) foreground onset times. We performed permutation tests to test for differences in the delay benefit across experiments (Experiment 6 versus Experiment 7 or Experiment 1 versus Experiment 7) by randomly shuffling participants across experiments and estimating the difference between the magnitude of improvement in each set of shuffled data. We repeated this procedure 10,000 times to build up a distribution of the test statistic (difference in delay benefit) under the null hypothesis (there is no difference across experiments) and calculated the p-value (two-tailed) as the proportion of times that absolute values from the null distribution were at least as large as the actual absolute difference in delay benefit between experiments. We performed an analogous permutation test to test for a difference in the timescale of improvement (quantified as the location of the elbow point) between Experiments 1 and 7.

To ensure that differences in the delay benefit were not driven by Experiment 6 having more participants with near-ceiling performance compared to Experiment 7, we ran a control analysis in which we selected groups of participants from each experiment to have similar asymptotic performance. To avoid errors of non-independence, we used data from foreground onset times of 1500, 2000, and 2500 ms to select the participant groups, and then measured the delay benefit using the data from the remaining foreground onset times for these participants. In practice, we found that naively matching asymptotic performance for the "selection" conditions (1500, 2000, and 2500 ms) did not result in fully matched performance for the held-out conditions (1250, 1750, and 2250 ms), presumably because the group selection criterion (i.e., the difference in performance between groups for the 1500, 2000, and 2500 ms conditions) had some contribution from noise, which left a residual difference in performance between groups in the held-out conditions. To minimize this difference in performance, we imposed a bias during the matching procedure and selected participant groups whose difference in performance in the selection conditions was as close as possible to the bias value. This bias value was determined by selecting the value which minimized the performance difference between groups via three-fold cross-validation across the three foreground onset times used as the selection conditions. In this way we obtained participant groups whose performance was approximately matched in independent data from the regime in which performance was asymptotic.

Experiment 8 (Repeating Background Noises on Alternate Trials)
Stimuli: The stimuli were identical to those of Experiment 6 but were sampled differently over the course of the experiment due to the constraints of the design (see Procedure below).

Procedure: For each subject, we randomly selected 8 of the 160 possible backgrounds to repeat on every other trial in blocks of 40 trials. On half of these trials, the background noise appeared in isolation. The other half of these trials contained a randomly selected foreground randomly assigned to one of the foreground onset time conditions such that each foreground onset time condition occurred once. For the remaining trials, we randomly selected 80 backgrounds to serve

as “non-repeating” trials. Each of these backgrounds appeared twice: once in isolation and once with a randomly selected foreground randomly assigned to one of the foreground onset time conditions. These non-repeating background trials were randomly ordered subject to the constraint that each foreground onset time condition (for the non-repeating backgrounds) occurred once during a block. Each background noise was a unique exemplar, and foregrounds were never repeated. The order of the blocks was chosen at random. On each trial, participants judged whether the stimulus contained one or two sound sources and were not explicitly informed that backgrounds would repeat.

Participants: A total of 528 participants were recruited through Prolific. Of these, 153 participants were excluded either because they failed the headphone check task, had self-reported hearing loss, withdrew from the experiment, completed less than 90% of experimental trials, or did not complete all critical trials at the start and end of each block. Finally, 7 participants were excluded due to low task performance (average d’ < 0.8). This resulted in a total of 368 participants included in data analyses. Of these participants, 181 identified as female, 178 as male, 5 as nonbinary (4 participants did not provide a response). The average age of participants was 38.3 (s.d.=12.7). All participants were unique to this experiment.

Sample size: We targeted the same sample size as in Experiment 6 (n=196) but multiplied it by two to account for the fact that there were half as many trials per condition (20 total conditions: 10 foreground onset times crossed with background type: repeated or non-repeated) in this experiment. Due to the nature of the screening procedure, the actual sample was slightly below the target sample size.

Statistics and data analysis: We calculated a hit rate for each of the 20 experimental conditions (10 foreground onset times crossed with 2 background types) and false alarm rates using the background-only trials for each background type (repeated or non-repeated), then quantified detection performance as d’. We performed a repeated measures ANOVA to analyze the effects of foreground onset time and background repetition on foreground detection performance. We assumed data was normally distributed and evaluated this by eye. Mauchly's test indicated that the assumption of sphericity had not been violated. For each main effect and interaction of interest, we reported F-statistics, p-values and $\eta^2_{partial}$. To test for a difference in the delay benefit between background types (repeated versus non-repeated), we performed a permutation test using the same procedure described above in Experiment 7. The control analysis matching asymptotic performance was also performed using the same procedure described above for Experiment 7. However, we note that performing this analysis for Experiment 8 necessitated using distinct but partially overlapping sets of participants for the repeated and non-repeated conditions, because the same participants completed both conditions in the original experiment.

Experiment 9 (Stationary Noise)
Stimuli: To create stationary noise backgrounds, we replaced each of the 160 texture backgrounds with spectrally matched noise. The spectrally matched noise was generated by setting the Fourier amplitudes of a noise signal equal to the Fourier amplitudes of the corresponding sound texture, randomizing the phases, and then performing the inverse Fourier transform. For each of the 160 foreground-background pairs, we constructed stimuli in which the foreground appeared at each of 10 possible temporal positions (foreground onset times of 250, 500, 750, 1000, 1250, 1500, 1750, 2000, 2250, and 2500 ms) and each of 2 possible SNRs (-6 and -10 dB). This yielded a total of 3200 stimuli containing a foreground sound. We also created an additional 160 stimuli that consisted of the stationary background noise only.

Procedure: The procedure was identical to that of Experiment 1.

Participants: A total of 294 participants were recruited through Prolific. Of these, 86 participants were excluded either because they failed the headphone check task, had self-reported hearing loss, withdrew from the experiment, or completed less than 90% of experimental trials. Finally, 3 participants were excluded due to low task performance (average d' < 0.8). This resulted in a total of 205 participants included in data analyses. Of these participants, 84 identified as female, 119 as male, and 2 as nonbinary. The average age of participants was 37.6 (s.d. = 12.3). All participants were unique to this experiment.

Sample size: We did not have pilot data for this experiment. Thus, we targeted double the sample size of Experiment 1 because of the possibility that the effect of foreground onset time might be smaller. Due to the nature of the screening procedure, we were slightly above the target sample size.

Statistics and data analysis: We calculated a hit rate for each of the 20 experimental conditions (10 foreground onset times crossed with 2 SNRs) and a single false alarm rate using the background-only trials, then quantified detection performance as d'. We performed a repeated measures ANOVA to analyze the effect of foreground onset time and SNR on foreground detection performance. We assumed data was normally distributed and evaluated this by eye. Mauchly's test indicated that the assumption of sphericity had not been violated. For each main effect and interaction of interest, we reported F-statistics, p-values and $\eta^2_{partial}$. To analyze the effect of stationarity on the pattern of foreground detection performance, we compared the results of Experiment 9 (more stationary spectrally matched noise backgrounds) to the pooled results of Experiments 1 and 7 (less stationary texture backgrounds; data from Experiments 1 and 7 were pooled to increase power given that they showed very similar results). To test for differences in both the magnitude and the timescale of improvement in foreground detection performance between background types (textures versus spectrally matched noises), we performed permutation tests using the same procedure described above in Experiment 7. The control analysis matching asymptotic performance was performed using the same procedure described above in Experiment 7.

Foreground-Background Similarity Analysis

Selection of stimulus pairings: We divided the foreground-background pairings from Experiment 7 (in which foregrounds and backgrounds were randomly paired) into two groups. The groups were selected to be matched in a measure of spectral difference between foreground and background, but to differ in the difference between foreground and background in a spectrotemporal filter basis. Specifically, for each foreground and background, we measured the mean excitation pattern as a summary measure of the spectrum. We also measured the power in each of the 541 PCA-derived spectrotemporal filters used in the observer model. Then, for each foreground-background pair, we computed the Euclidean distance between the excitation patterns and between the spectrotemporal filter powers for the two sounds. Because the two distances are on different scales, we performed min-max normalization for each to scale them between 0 and 1. Next, to select pairings, we calculated the ratio of the spectrotemporal distance to the spectral distance. This ratio is largest when the spectrotemporal distance is large and the spectral distance is small. We then used this measure to split pairings into two groups (low and high spectrotemporal similarity) subject to the constraint that the two groups of pairings contained the same number of occurrences of each foreground and background. This ensures that the results we see are due to the pairings rather than to differences in the specific foregrounds or backgrounds in the two stimulus groups. The free parameter in this procedure was the proportion of total pairings included in the two groups (including all pairings maximized the number of pairings in each group, but led to a smaller difference between groups than if not all pairings were included.

We opted to use 75% of all possible pairings, discarding the middle 25% of pairings surrounding the median spectrotemporal distance. This yielded groups containing 9,600 pairings. Because participants were presented with randomly chosen pairings, not all of these 9,600 pairings had been presented to participants (6,923 of the large spectrotemporal distance group and 6,854 of the small spectrotemporal distance group were actually used in the experiment). Figure S5 plots the results separately for trials whose stimuli fell into one group or the other.

Statistics and data analysis: Re-analyzing the data from Experiment 7, we calculated a hit rate for each of the 2 groups of pairings for each of the 10 foreground onset times. Using the false alarm rate from the background-only trials of Experiment 7, we quantified detection performance as d'. We performed a repeated measures ANOVA to analyze the effect of foreground onset time and foreground-background similarity on foreground detection performance. We assumed data was normally distributed and evaluated this by eye. Mauchly's test indicated that the assumption of sphericity had not been violated. For each main effect and interaction of interest, we reported F-statistics, p-values and $\eta^2_{partial}$. To test for differences in the timescale of improvement in foreground detection performance between pairing types (more similar versus less similar), we performed permutation tests using the same procedure described above for Experiment 7.

Experiment 10 (Harmonic Foregrounds)
Stimuli: To obtain a set of (approximately) harmonic foreground sounds, we selected human vocalizations and musical instrument sounds from a dataset of isolated sound events (GISE-51 training set (21)). Specifically, we selected human vocalization sounds from the "human_speech", "laughter" and "screaming" categories and selected musical instrument sounds from the "gong", "guitar", "harmonica", "harp", "marimba_and_xylophone", "organ", "piano", and "trumpet" categories. For each sound in the training set category, we used YIN (22) to measure the average periodicity (one minus aperiodicity) in a sliding 0.5 s window, discarding windowed segments that were mostly quiet or were outside of a periodicity range of 0.9 to 0.99. We set the lower bound to ensure selected sounds would be highly periodic and set the upper bound because windowed segments whose periodicity was greater than 0.99 tended to be tones (e.g., dial tones present in clips labeled as "human_speech") rather than speech or musical instruments. From the windowed segments of a given sound, we selected the segment with the maximum periodicity (i.e., the most harmonic segment). This resulted in a single 0.5 s clip for each sound in the training set categories described above. Finally, we removed any sounds that were near duplicates (by measuring the power in a set of spectrotemporal filters, computing the correlation of spectrotemporal filter power across sounds, and removing sounds whose correlation exceeded 0.8) or had an estimated F0 below 100 Hz or above 1000 Hz. From the sounds that remained, we chose the most periodic from each category, selecting 40 examples of human speech, 20 examples of laughter and screaming each, as well as 10 examples from each musical instrument category (see Table S3 for a list of the selected sounds).

Procedure: The procedure was identical to that of Experiment 7.

Participants: A total of 485 participants were recruited through Prolific. Of these, 193 participants were excluded either because they failed the headphone check task, had self-reported hearing loss, withdrew from the experiment, or completed less than 90% of experimental trials. Finally, 5 participants were excluded due to low task performance (average d' < 0.8). This resulted in a total of 287 participants included in data analyses. Of these participants, 127 identified as female, 151 as male, and 3 as nonbinary (6 participants did not provide a response). The average age of participants was 32.2 (s.d. = 9.7). All participants were unique to this experiment.

Sample size: Because we did not have pilot data for this experiment, we initially targeted a sample size double that of Experiment 1 to account for the possibility that the effect of foreground onset time might be smaller. However, we found it difficult to reliably fit elbow functions to this data because the effect of foreground onset time was so small. Thus, we increased the sample size by about 50% to improve the reliability of the elbow function fits to this data.

Statistics and data analysis: We calculated a hit rate for each of the 10 experimental conditions (10 foreground onset times) and a single false alarm rate using the background-only trials, then quantified detection performance as d'. We performed a repeated measures ANOVA to analyze the effect of foreground onset time on foreground detection performance. We assumed data was normally distributed and evaluated this by eye. Mauchly's test indicated that the assumption of sphericity had not been violated. For each main effect and interaction of interest, we reported F-statistics, p-values and $\eta^2_{partial}$. To analyze the effect of harmonicity on the pattern of foreground detection performance, we compared the results of Experiment 10 (harmonic foregrounds) to the pooled results of Experiments 1 and 7 (less harmonic foregrounds; data from Experiments 1 and 7 were pooled to increase power given that they showed very similar results). To test for differences in both the magnitude and the timescale of improvement in foreground detection performance between foreground types, we performed permutation tests using the same procedure described above in Experiment 7. The control analysis matching asymptotic performance was also performed using the same procedure described above in Experiment 7.

SI Figures & Tables

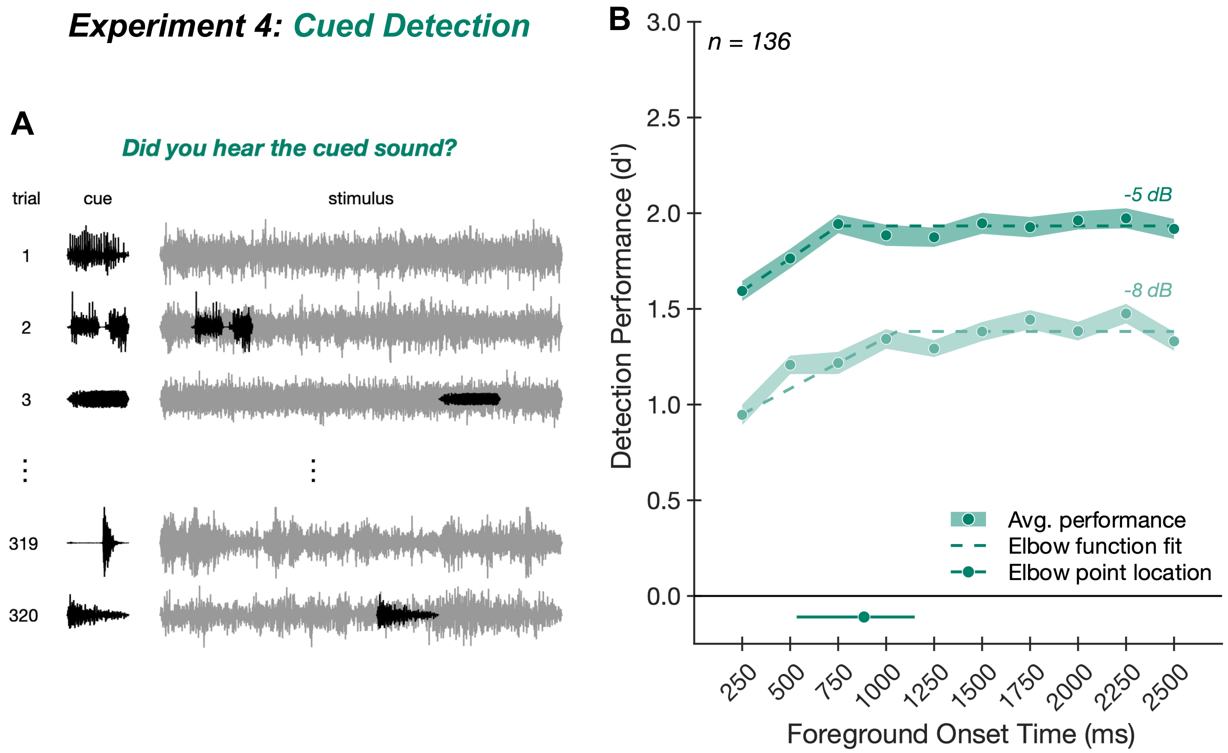

Figure S1. Experiment 4: Benefit of background exposure persists despite knowing what to listen for.

(*A*) Experiment 4 task. On each trial, participants first heard a foreground sound in isolation (left, black), followed by continuous background noise (right, gray). Half of the trials contained the cued sound superimposed on the background (e.g., trial 2), and participants judged whether the stimulus contained the cued sound. Because detection performance typically benefits from knowing what to listen for (23), we reduced the SNR of the foreground relative to the background to approximately match the level of performance observed in Experiment 1. (*B*) Experiment 4 results. Average foreground detection performance (quantified as d'; green circles) is plotted as a function of SNR and foreground onset time. Shaded regions plot standard errors. Dashed lines plot elbow function fit. Solid line below main axis plots one standard deviation above and below the median elbow point, obtained by fitting elbow functions to the results averaged over SNR and bootstrapping over participants; dot on this line plots the fitted elbow point from the complete participant sample.

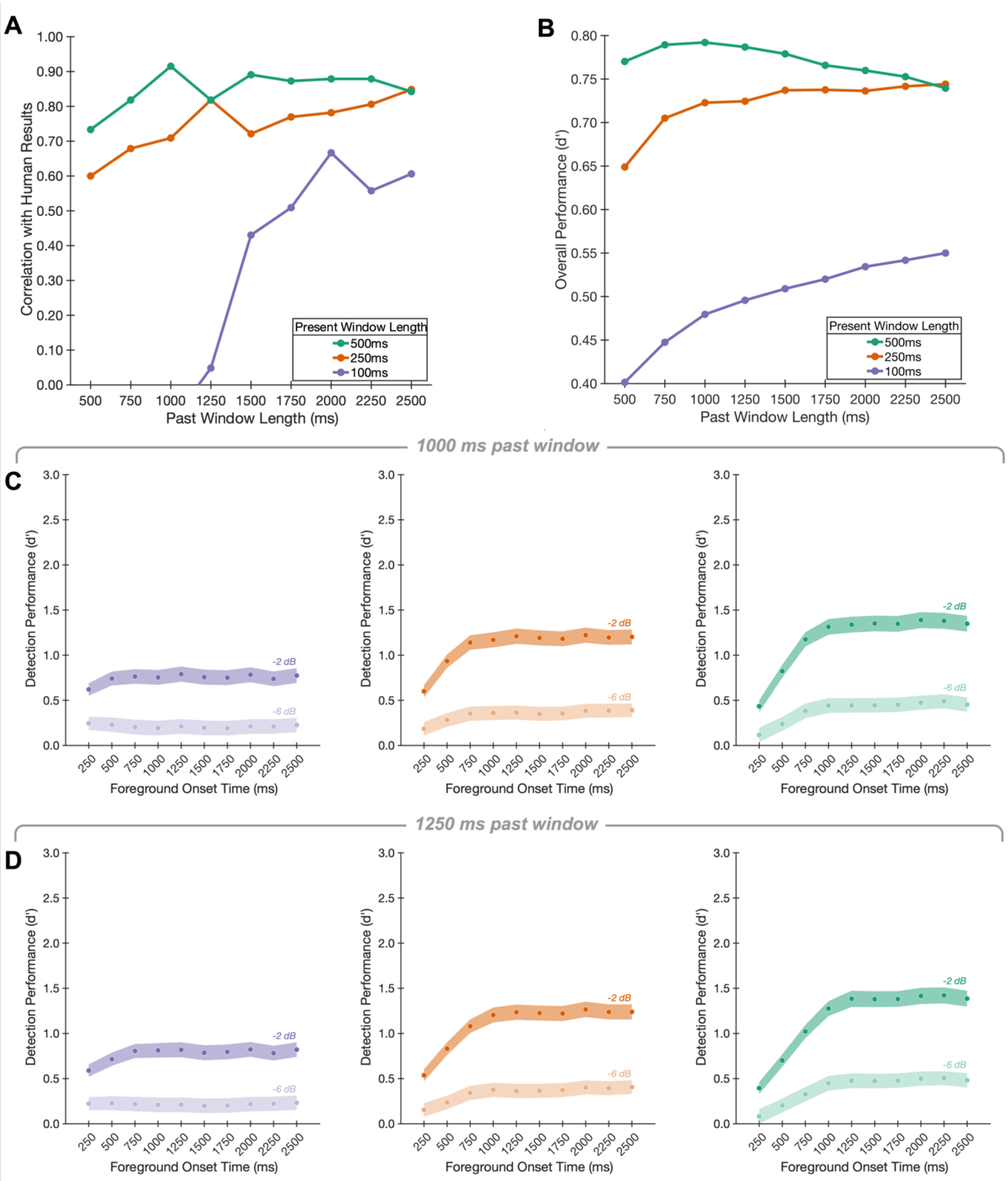

Figure S2. Observer model results for different window sizes.

(*A*) Human-model correlations for different window sizes. The Spearman correlation between the model results and the human results from Experiment 1 is plotted as a function of past window length for each present window length (100ms in purple, 250 ms in orange, and 500 ms in green). (*B*) Overall model performance for different window sizes. The overall model performance (computed by averaging detection performance over SNR and foreground onset time) is plotted as a function of past window length for each present window length. Same conventions as *A*. (*C*) Model results using 1000 ms past window. Each panel plots model foreground detection performance as a function of SNR and foreground onset time for a different present window length (100ms left, purple; 250 ms middle, orange; 500 ms right, green) using a fixed past window length of 1000 ms. Shaded regions plot standard deviations of performance obtained by bootstrapping over stimuli. (*D*) Model results using 1250 ms past window. Same conventions as *C* but using a fixed past window length of 1250 ms.

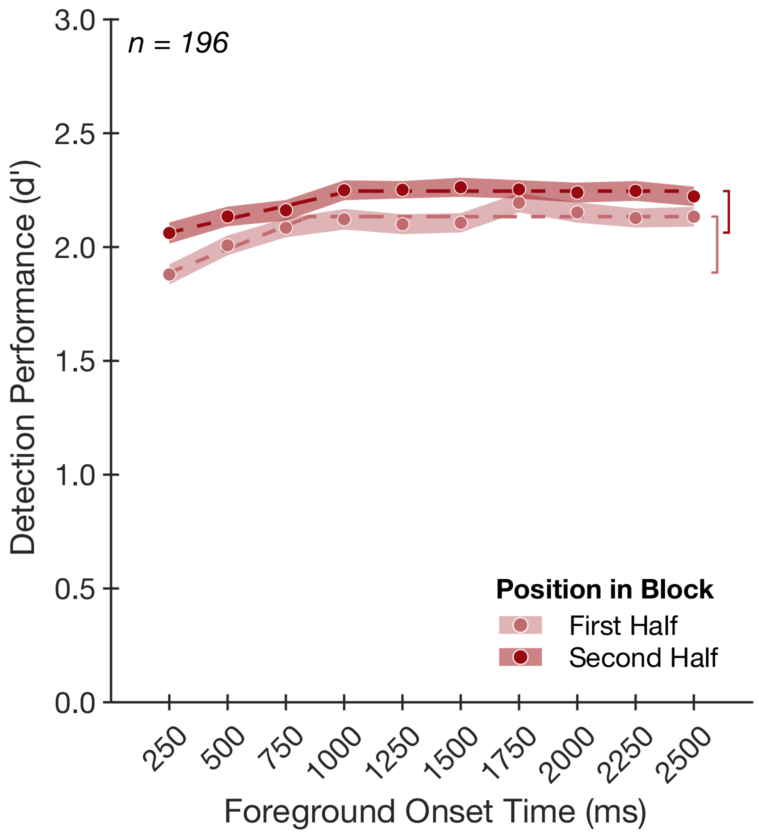

Figure S3. Experiment 6: Repetition of background noise enhances foreground detection. Average foreground detection performance (quantified as d') is plotted as a function of foreground onset time for the first half of trials (light red circles) versus the second half of trials (dark red circles) within a block. Shaded regions plot standard errors. Dashed lines plot elbow function fit. Vertical brackets denote the delay benefit.

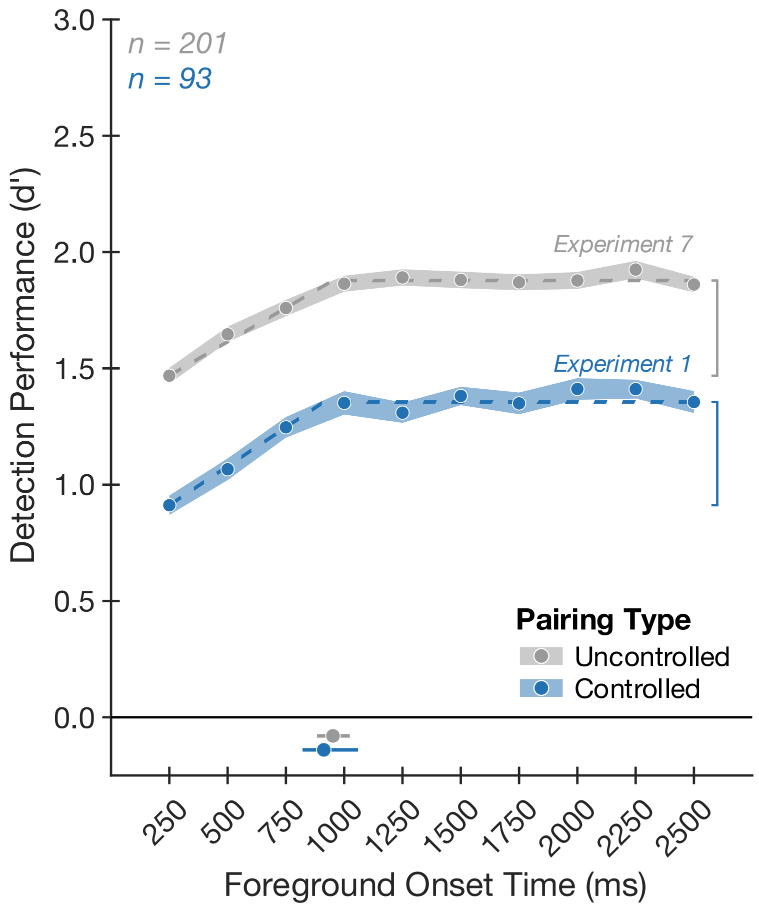

Figure S4. Experiments 1 & 7: Benefit of exposure to background noise is unaffected by choice of foreground-background pairings.

Average foreground detection performance (quantified as d') is plotted as a function of foreground onset time for Experiment 1 (controlled foreground-background pairings; blue circles) versus Experiment 7 (uncontrolled foreground-background pairings; gray circles). Shaded regions plot standard errors. Dashed lines plot elbow function fit. Solid lines below main axis plot one standard deviation above and below the median elbow points (Experiment 1 shown in blue; Experiment 7 shown in gray), obtained by fitting elbow functions to the results averaged over SNR and bootstrapping over participants; dots on these lines plot the fitted elbow points from the complete participant samples. Vertical brackets denote the delay benefit.

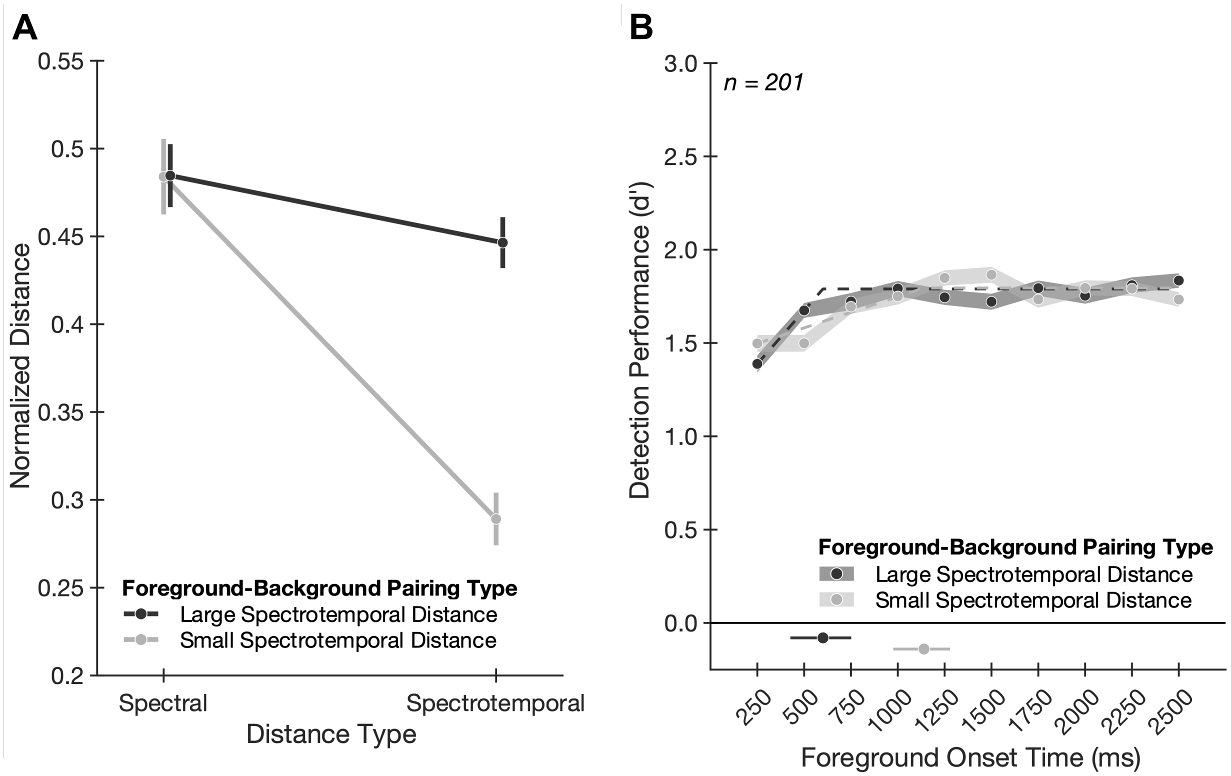

Figure S5. Effect of background exposure depends on foreground-background similarity.
(*A*) Spectral and spectrotemporal similarity of foreground-background pairs. The average normalized distance between foregrounds and backgrounds is plotted as a function of distance type for two groups of foreground-background pairings. The two groups of pairings were approximately matched in spectral distance but differed in spectrotemporal distance. Error bars plot standard deviations. (*B*) Foreground-background similarity results. Average foreground detection performance (quantified as d') is plotted as a function of foreground onset time for the two groups of foreground-background pairs. Shaded regions plot standard errors. Dashed lines plot elbow function fit. Solid lines below main axis plot one standard deviation above and below the median elbow points (more similar pairs shown in gray; less similar pairs shown in black), obtained by bootstrapping over participants; dots on these lines plot the fitted elbow points from the complete participant samples.

Table S1. AudioSet labels that were excluded in the process of obtaining texture sounds from which the background noises were drawn.

Speech	
0,/m/09x0r,"Speech"	13,/t/dd00135,"Children shouting"
1,/m/05zppz,"Male speech, man speaking"	15,/m/02rtxlg,"Whispering"
2,/m/02zsn,"Female speech, woman speaking"	27,/m/015lz1,"Singing"
3,/m/0ytgt,"Child speech, kid speaking"	28,/m/0l14jd,"Choir"
4,/m/01h8n0,"Conversation"	29,/m/01swy6,"Yodeling"
5,/m/02qldy,"Narration, monologue"	30,/m/02bk07,"Chant"
6,/m/0261r1,"Babbling"	31,/m/01c194,"Mantra"
7,/m/0brhx,"Speech synthesizer"	32,/t/dd00003,"Male singing"
8,/m/07p6fty,"Shout"	33,/t/dd00004,"Female singing"
9,/m/07q4ntr,"Bellow"	34,/t/dd00005,"Child singing"
10,/m/07rwj3x,"Whoop"	35,/t/dd00006,"Synthetic singing"
11,/m/07sr1lc,"Yell"	36,/m/06bxc,"Rapping"
12,/m/04gy_2,"Battle cry"	
Music	
137,/m/04rlf,"Music"	210,/m/0192l,"Bagpipes"
138,/m/04szw,"Musical instrument"	211,/m/02bxd,"Didgeridoo"
139,/m/0fx80y,"Plucked string instrument"	212,/m/0l14l2,"Shofar"
140,/m/0342h,"Guitar"	213,/m/07kc_,"Theremin"
141,/m/02sgy,"Electric guitar"	214,/m/0l14t7,"Singing bowl"
142,/m/018vs,"Bass guitar"	215,/m/01hgjl,"Scratching (performance technique)"
143,/m/042v_gx,"Acoustic guitar"	216,/m/064t9,"Pop music"
144,/m/06w87,"Steel guitar, slide guitar"	217,/m/0glt670,"Hip hop music"
145,/m/01glhc,"Tapping (guitar technique)"	218,/m/02cz_7,"Beatboxing"
146,/m/07s0s5r,"Strum"	219,/m/06by7,"Rock music"
147,/m/018j2,"Banjo"	220,/m/03lty,"Heavy metal"
148,/m/0jtg0,"Sitar"	221,/m/05r6t,"Punk rock"
149,/m/04rzd,"Mandolin"	222,/m/0dls3,"Grunge"
150,/m/01bns_,"Zither"	223,/m/0dl5d,"Progressive rock"
151,/m/07xzm,"Ukulele"	224,/m/07sbbz2,"Rock and roll"
152,/m/05148p4,"Keyboard (musical)"	225,/m/05w3f,"Psychedelic rock"
153,/m/05r5c,"Piano"	226,/m/06j6l,"Rhythm and blues"
154,/m/01s0ps,"Electric piano"	227,/m/0gywn,"Soul music"
155,/m/013y1f,"Organ"	228,/m/06cqb,"Reggae"
156,/m/03xq_f,"Electronic organ"	229,/m/01lyv,"Country"
157,/m/03gvt,"Hammond organ"	230,/m/015y_n,"Swing music"
158,/m/0l14qv,"Synthesizer"	231,/m/0gg8l,"Bluegrass"
159,/m/01v1d8,"Sampler"	232,/m/02x8m,"Funk"
160,/m/03q5t,"Harpsichord"	233,/m/02w4v,"Folk music"
161,/m/0l14md,"Percussion"	234,/m/06j64v,"Middle Eastern music"
162,/m/02hnl,"Drum kit"	235,/m/03_d0,"Jazz"
163,/m/0cfdd,"Drum machine"	236,/m/026z9,"Disco"
164,/m/026t6,"Drum"	237,/m/0ggq0m,"Classical music"
165,/m/06rvn,"Snare drum"	238,/m/05lls,"Opera"
166,/m/03t3fj,"Rimshot"	239,/m/02lkt,"Electronic music"
167,/m/02k_mr,"Drum roll"	240,/m/03mb9,"House music"
168,/m/0bm02,"Bass drum"	241,/m/07gxw,"Techno"
169,/m/011k_j,"Timpani"	242,/m/07s72n,"Dubstep"
170,/m/01p970,"Tabla"	243,/m/0283d,"Drum and bass"

171,/m/01qbl,"Cymbal"	244,/m/0m0jc,"Electronica"
172,/m/03qtq,"Hi-hat"	245,/m/08cyft,"Electronic dance music"
173,/m/01sm1g,"Wood block"	246,/m/0fd3y,"Ambient music"
174,/m/07brj,"Tambourine"	247,/m/07lnk,"Trance music"
175,/m/05r5wn,"Rattle (instrument)"	248,/m/0g293,"Music of Latin America"
176,/m/0xzly,"Maraca"	249,/m/0ln16,"Salsa music"
177,/m/0mbct,"Gong"	250,/m/0326g,"Flamenco"
178,/m/016622,"Tubular bells"	251,/m/0155w,"Blues"
179,/m/0j45pbj,"Mallet percussion"	252,/m/05fw6t,"Music for children"
180,/m/0dwsp,"Marimba, xylophone"	253,/m/02v2lh,"New-age music"
181,/m/0dwtp,"Glockenspiel"	254,/m/0y4f8,"Vocal music"
182,/m/0dwt5,"Vibraphone"	255,/m/0z9c,"A capella"
183,/m/0l156b,"Steelpan"	256,/m/0164x2,"Music of Africa"
184,/m/05pd6,"Orchestra"	257,/m/0145m,"Afrobeat"
185,/m/01kcd,"Brass instrument"	258,/m/02mscn,"Christian music"
186,/m/0319l,"French horn"	259,/m/016cjb,"Gospel music"
187,/m/07gql,"Trumpet"	260,/m/028sqc,"Music of Asia"
188,/m/07c6l,"Trombone"	261,/m/015vgc,"Carnatic music"
189,/m/0l14_3,"Bowed string instrument"	262,/m/0dq0md,"Music of Bollywood"
190,/m/02qmj0d,"String section"	263,/m/06rqw,"Ska"
191,/m/07y_7,"Violin, fiddle"	264,/m/02p0sh1,"Traditional music"
192,/m/0d8_n,"Pizzicato"	265,/m/05rwpb,"Independent music"
193,/m/01xqw,"Cello"	266,/m/074ft,"Song"
194,/m/02fsn,"Double bass"	267,/m/025td0t,"Background music"
195,/m/085jw,"Wind instrument, woodwind instrument"	268,/m/02cjck,"Theme music"
196,/m/0l14j_,"Flute"	269,/m/03r5q_,"Jingle (music)"
197,/m/06ncr,"Saxophone"	270,/m/0l14gg,"Soundtrack music"
198,/m/01wy6,"Clarinet"	271,/m/07pkxdp,"Lullaby"
199,/m/03m5k,"Harp"	272,/m/01z7dr,"Video game music"
200,/m/0395lw,"Bell"	273,/m/0140xf,"Christmas music"
201,/m/03w41f,"Church bell"	274,/m/0ggx5q,"Dance music"
202,/m/027m70_,"Jingle bell"	275,/m/04wptg,"Wedding music"
203,/m/0gy1t2s,"Bicycle bell"	276,/t/dd00031,"Happy music"
204,/m/07n_g,"Tuning fork"	277,/t/dd00032,"Funny music"
205,/m/0f8s22,"Chime"	278,/t/dd00033,"Sad music"
206,/m/026fgl,"Wind chime"	279,/t/dd00034,"Tender music"
207,/m/0150b9,"Change ringing (campanology)"	280,/t/dd00035,"Exciting music"
208,/m/03qjg,"Harmonica"	281,/t/dd00036,"Angry music"
209,/m/0mkg,"Accordion"	282,/t/dd00037,"Scary music"
Sourceless	
500,/m/028v0c,"Silence"	503,/m/0hdsk,"Chirp tone"
501,/m/01v_m0,"Sine wave"	504,/m/0c1dj,"Sound effect"
502,/m/0b9m1,"Harmonic"	505,/m/07pt_g0,"Pulse"

Table S2. Foreground-background pairings used in Experiments 1-5 and 9. Experiments 6-8 used the same set of foregrounds and backgrounds but paired randomly. Experiment 10 used the same backgrounds but different foregrounds (see Table S3).

#	*Foreground*	*Background (YouTube ID)*	#	*Foreground*	*Background (YouTube ID)*
1	cuckoo clock	f4BDxEci8Nk	81	rooster	BS_RcUnRB_g
2	cutting scissors	DNuH5JyeF28	82	running hard surface	4ZqibG97oXc
3	dentist drill	ujNf-Q9q8BQ	83	running gravel	uREJNYW9Zgc
4	dial tone	ul285Kv4Zzw	84	run up stairs	ZwFsvLQoqhw
5	dialing	auW7P6Fla1s	85	blacksmith hammering	RhYlsWU4xkA
6	rotary dialer	LrP3e_XDEn0	86	saltshaker	WGJvqPhmbx8
7	dialup	7CSwogGrVXE	87	sander	k7JefMIMghA
8	dice roll	HgmZlVe3W18	88	school bell	XoG5PD231R4
9	dish clanking	YkxxnlvB3mk	89	screw lid off	C9lHMPdX_3w
10	dog barking	oDQuFo9Yl60	90	seagull	EQW7Tq1TpTA
11	dog drinking	0CahCF_csFE	91	seal	1lsFTFuLmtw
12	dog whining	u-oxLds6JvI	92	glass shattering	0SsaL_YNyjY
13	door open	1pSbmqnIrb8	93	blowing on bottle	j4AAuB9XLVA
14	door squeak	mlEy1yQpUYs	94	sheep	DOIX0j6Eg98
15	doorbell	Q8G3Y_b3GKc	95	shuffling	pJGThhDN_ZQ
16	dove cooing	d9lZjw66ow0	96	sipping	J8dsyhhvRO0
17	dreidel spinning	9amjTzhfO_0	97	siren	FqigDul5Mts
18	drawer close	MiEfLgS0v6k	98	sleighbells	uYXXcFA8NXE
19	drawer opening	k-VZj_uYyjg	99	slicing	PicbqGLxpbM
20	drinking	7A14Op2mCFQ	100	slot machine	vSFbqLgy3hA
21	dripping	Kc3-d4yhnr4	101	birdsong	OE94H63nLOw
22	bass drum	MRz22sll8Ko	102	splash water	eTaaBEx8Uz0
23	duck quack	F1j9Fleij8g	103	stapling	17XCtcwI74Y
24	handheld drill	GyaYw1BQ7tE	104	space ship door	5eO0cJNgV74
25	elevator door	f36dN2cr4j8	105	sword fighting	yPTe-JL28xU
26	faucet	J0kO9X49clQ	106	tap dancing	XoENpj5z6Oc
27	fax	-3KtNOqHb2A	107	telephone ring	WXT0aIPTRXM
28	balloon deflating	Xlr0Maz4jvI	108	tennis rally	ChDKSOopmUs
29	finger tapping	14gMYPgWBk0	109	toy squeak	tU5X5sCZj3U
30	fire alarm	w41FKuP0HT8	110	train warning bell	-mQyAYU_Bd4
31	fireworks	1kBruu0XiCk	111	trampoline	DCHJSNDvkYs
32	foghorn	PZ4j2qcmvGk	112	bowling	BOeA1ya8Bwk
33	frog croak	-e_wUbIdJuk	113	triangle	Cyi_IJ2Z4Yc
34	gavel	oA96zEIi58o	114	truck backup beeping	TVX2OXNhvE4
35	geese honking	AhG32tIdaaE	115	turkey gobble	6VcCuP4YeU4
36	gong	vS6ERMn4_58	116	typing	IDg45ezSvZE
37	gorilla	PBuW6_LuAAk	117	walking hard surface	Kl5tat_bJio
38	grandfather clock	O9gqbLxkA7I	118	walking with heels	Yrz6NzBsoOk
39	gunshots	bcU49USpFcY	119	whale call	u2o-xqEvKUk
40	hammer	O_kxjfU50lc	120	wolves howling	Cy2zzRtUDDI
41	dribble	Nf0K0I00WMY	121	writing chalkboard	z_cfAqGxyT4
42	hawk screech	CHEJS1BA0_0	122	writing on whiteboard	iOfxRUARYBQ
43	horse neighing	yCvz0W1Spv8	123	zipper	7aoxC_WRv3I
44	ice machine	8K6acvCdQqU	124	crack nuts	J4gtEa7HC3s
45	jumping rope	IXljCR3iqLY	125	mac startup	63USLppGyX8
46	kazoo	4EyYLQ9QQLY	126	noisemaker	aDvPcdwm0_8
47	kettle	PBe8eexve2Q	127	peeling potato	VNos6WvqE-o
48	key opening door	tshdgKCaea8	128	radar beeps	DmDPTWAChc0

49	keys jingling	JjkxEDKt_WI		129	scrubbing dishes	BgONm1BKM68
50	knocking on door	pDppR23BSrE		130	sports arena buzzer	DK7CYXsz8TI
51	knuckle cracking	UAidYn4tDuQ		131	busy signal	nIeOtZISuVw
52	laser gun	_9roxFqE_0s		132	windows startup sound	PSkAi5SysFQ
53	lion growl	pGx04X2S8Vo		133	windup toy	X9hEru96BO0
54	locker closing	Zb4c7wX7MGc		134	air hockey	ZumNxD30rO8
55	locker opening	-x5fDrtsR-M		135	camera taking picture	_eyfihZLgkE
56	bear growling	gYUb7ZEx2ig		136	heart beat	LjnfmSFcv8I
57	morse code	22rlvtV9tjY		137	owl hooting	873MpPwYc0s
58	nose blowing	X9HsMWDHhDs		138	battle sounds	GfiRcVgqRhk
59	reception desk bell	1DKHkGt6A5Y		139	monkey scream	C5oSLRvLVOA
60	bike bell	6UDbuFKXaR0		140	car alarm	VXpimpAohoE
61	pager beeps	XXmxZdWEfDU		141	car crash	YRixc_jc9S8
62	paper cutter	8FNGAEJom4k		142	alarm clock	qlJ1JZ5chP8
63	paper shredder	eY-Z97PApDo		143	car horn	Jdm3aEGx5LI
64	parrot	MDs84SJcUIl		144	car wheels skidding	YeghkUAmB7I
65	pepper grinder	NTWLwrQZ2xA		145	cash register	3LKkiKyWqlQ
66	pet collar jingling	VZwHbQCLeBY		146	castanet	M4MWeVg6Rh0
67	pig snort	vsOjgzS4ZF8		147	cat meow	eGE475m0FcE
68	pinball	Xwg3gNWSg1U		148	cat purring	jNWjIt-qjAs
69	ping pong	J_qoxB9ytXY		149	alien sound effects	CR_pvgUzL5M
70	pool ball	M9i6UvtYtpA		150	phone vibrate	e5Qk-uZSDQ0
71	popcorn popping	uPYXAScbHt8		151	chainsaw revving	iP5TKSoB3-Y
72	pour out bottle	OF7A3vsntYk		152	chair being raised	7xfgJ9VVGdo
73	printer startup	wfHeoPDLMaM		153	chicken cluck	_Habb1lwnPM
74	printing	WjCndbbxq54		154	wind chimes	KORQwq2m20I
75	ratchet	TGNzh4wMT0k		155	chopping wood	RB28Vj9j-yY
76	crow	nH5SVpHQRRs		156	clipping hair	IaCX2UnQZAE
77	whistle	cIUorSTEISM		157	coins dropping	TL7iX2d72Y0
78	rock fall water	c6m6ZPOnAJ0		158	coin vending machine	4IEpPBzMCN4
79	rock fall ground	JOyDnPX8WRk		159	cow moo	SU3VCol6mSY
80	rocking chair	gUJzqny-Ph0		160	ATM	Qd1RHwg2Duw

Table S3. Foregrounds used in Experiment 10, taken from GISE-51 training set

#	Category	Stimulus ID	#	Category	Stimulus ID
1	Human_speech	107100	81	Gong	209917_filtered
2	Human_speech	92940	82	Gong	222192
3	Human_speech	80641	83	Gong	57389
4	Human_speech	63334	84	Gong	222191
5	Human_speech	181948_filtered	85	Gong	57419
6	Human_speech	106439	86	Gong	206023
7	Human_speech	32168	87	Gong	57424
8	Human_speech	93787_filtered	88	Gong	222923_filtered
9	Human_speech	91076	89	Gong	222217
10	Human_speech	278944	90	Gong	222922_filtered
11	Human_speech	95238	91	Guitar	128844_filtered
12	Human_speech	77409	92	Guitar	40438
13	Human_speech	109838_filtered	93	Guitar	251187_filtered
14	Human_speech	82710	94	Guitar	64791_filtered
15	Human_speech	50497_filtered	95	Guitar	251161_filtered
16	Human_speech	216567	96	Guitar	28399
17	Human_speech	108875	97	Guitar	251156_filtered
18	Human_speech	92951	98	Guitar	48355
19	Human_speech	92961	99	Guitar	252448
20	Human_speech	18287	100	Guitar	18474
21	Human_speech	77416	101	Harmonica	116903_filtered
22	Human_speech	235110	102	Harmonica	116897_filtered
23	Human_speech	77421	103	Harmonica	116902
24	Human_speech	184432_filtered	104	Harmonica	325376
25	Human_speech	92941	105	Harmonica	330759_filtered
26	Human_speech	371581	106	Harmonica	116887_filtered
27	Human_speech	235114	107	Harmonica	325377
28	Human_speech	422718_filtered	108	Harmonica	116874_filtered
29	Human_speech	104707	109	Harmonica	116875_filtered
30	Human_speech	92965	110	Harmonica	116869
31	Human_speech	109865	111	Harp	53854
32	Human_speech	104728	112	Harp	53867
33	Human_speech	107099	113	Harp	53846
34	Human_speech	77413	114	Harp	53864
35	Human_speech	67627	115	Harp	373563_filtered
36	Human_speech	106428	116	Harp	53866
37	Human_speech	181316	117	Harp	415387
38	Human_speech	106440	118	Harp	415463
39	Human_speech	77412	119	Harp	415433
40	Human_speech	92919	120	Harp	415466
41	Laughter	132812	121	Marimba_and_xylophone	373585_filtered
42	Laughter	364485_filtered	122	Marimba_and_xylophone	202584
43	Laughter	171707	123	Marimba_and_xylophone	216753_filtered
44	Laughter	95814	124	Marimba_and_xylophone	202576
45	Laughter	174625	125	Marimba_and_xylophone	216755_filtered
46	Laughter	411082	126	Marimba_and_xylophone	216761_filtered
47	Laughter	198263	127	Marimba_and_xylophone	216752_filtered
48	Laughter	266004	128	Marimba_and_xylophone	202577
49	Laughter	57734	129	Marimba_and_xylophone	216751_filtered
50	Laughter	172923_filtered	130	Marimba_and_xylophone	216756_filtered
51	Laughter	132809_filtered	131	Organ	245073

52	Laughter	364444_filtered		132	Organ	373682_filtered
53	Laughter	95816		133	Organ	160297
54	Laughter	62257_filtered		134	Organ	11856
55	Laughter	198252_filtered		135	Organ	373685_filtered
56	Laughter	132746_filtered		136	Organ	373688
57	Laughter	393342_filtered		137	Organ	160301
58	Laughter	366173		138	Organ	373691_filtered
59	Laughter	196066		139	Organ	373687_filtered
60	Laughter	9557		140	Organ	333753
61	Screaming	219665		141	Piano	148539_filtered
62	Screaming	163729_filtered		142	Piano	11655_filtered
63	Screaming	220665		143	Piano	173763_filtered
64	Screaming	219663		144	Piano	351928
65	Screaming	59163		145	Piano	11656_filtered
66	Screaming	131709		146	Piano	382533_filtered
67	Screaming	219666_filtered		147	Piano	32019_filtered
68	Screaming	9434_filtered		148	Piano	11644_filtered
69	Screaming	58794		149	Piano	83132
70	Screaming	104035		150	Piano	11626_filtered
71	Screaming	222586		151	Trumpet	374138_filtered
72	Screaming	351630		152	Trumpet	357321_filtered
73	Screaming	104028_filtered		153	Trumpet	357560_filtered
74	Screaming	261853_filtered		154	Trumpet	247248_filtered
75	Screaming	166154_filtered		155	Trumpet	247220_filtered
76	Screaming	42849_filtered		156	Trumpet	247276_filtered
77	Screaming	221151		157	Trumpet	357419
78	Screaming	104033		158	Trumpet	247216_filtered
79	Screaming	220291		159	Trumpet	247110
80	Screaming	61219		160	Trumpet	247381

SI References

1. A. Francl, J. H. McDermott, Deep neural network models of sound localization reveal how perception is adapted to real-world environments. *Nat. Hum. Behav.* **6**, 111–133 (2022).
2. S. Norman-Haignere, N. G. Kanwisher, J. H. McDermott, Distinct cortical pathways for music and speech revealed by hypothesis-free voxel decomposition. *Neuron* (2015). https://doi.org/10.1016/j.neuron.2015.11.035.
3. M. J. McPherson, R. C. Grace, J. H. McDermott, Harmonicity aids hearing in noise. *Attention, Perception, Psychophys.* **84**, 1016–1042 (2022).
4. J. F. Gemmeke, *et al.*, Audio Set: An ontology and human-labeled dataset for audio events in *ICASSP, IEEE International Conference on Acoustics, Speech and Signal Processing - Proceedings*, (2017), pp. 776–780.
5. R. McWalter, J. H. McDermott, Adaptive and selective time averaging of auditory scenes. *Curr. Biol.* **28**, 1405-1418.e10 (2018).
6. A. J. E. Kell, J. H. McDermott, Invariance to background noise as a signature of non-primary auditory cortex. *Nat. Commun.* **10**, 1–11 (2019).
7. R. McWalter, J. H. McDermott, Illusory sound texture reveals multi-second statistical completion in auditory scene analysis. *Nat. Commun.* **10** (2019).
8. J. H. McDermott, E. P. Simoncelli, Sound texture perception via statistics of the auditory periphery: Evidence from sound synthesis. *Neuron* **71**, 926–940 (2011).
9. J. Feather, “Evaluating machine learning models of sensory systems.” (2023).
10. H. W. Kuhn, The Hungarian method for the assignment problem. *Nav. Res. Logist. Q.* **2**, 83–97 (1955).
11. K. J. P. Woods, J. H. McDermott, Schema learning for the cocktail party problem. *Proc. Natl. Acad. Sci. U. S. A.* **115**, E3313–E3322 (2018).
12. A. J. E. Kell, D. L. K. Yamins, E. N. Shook, S. V. Norman-Haignere, J. H. McDermott, A task-optimized neural network replicates human auditory behavior, predicts brain responses, and reveals a cortical processing hierarchy. *Neuron* **98**, 630-644.e16 (2018).
13. M. J. McPherson, J. H. McDermott, Time-dependent discrimination advantages for harmonic sounds suggest efficient coding for memory. *Proc. Natl. Acad. Sci. U. S. A.* **117**, 32169–32180 (2020).
14. M. J. McPherson, *et al.*, Perceptual fusion of musical notes by native Amazonians suggests universal representations of musical intervals. *Nat. Commun.* **11** (2020).
15. J. Traer, S. V. Norman-Haignere, J. H. McDermott, Causal inference in environmental sound recognition. *Cognition* **214** (2021).
16. K. J. P. Woods, M. H. Siegel, J. Traer, J. H. McDermott, Headphone screening to facilitate web-based auditory experiments. *Attention, Perception, Psychophys.* (2017). https://doi.org/10.3758/s13414-017-1361-2.
17. R. Bianco, G. Mills, M. de Kerangal, S. Rosen, M. Chait, Reward enhances online participants’ engagement with a demanding auditory task. *Trends Hear.* **25** (2021).
18. F. Faul, E. Erdfelder, A. Buchner, A.-G. Lang, Statistical power analyses using G* Power 3.1: Tests for correlation and regression analyses. *Behav. Res. Methods* **41**, 1149–1160 (2009).
19. B. R. Glasberg, B. C. J. Moore, Derivation of auditory filter shapes from notched-

noise data. *Hear. Res.* **47**, 103–138 (1990).
20. C. P. Lanting, P. M. Briley, C. J. Sumner, K. Krumbholz, Mechanisms of adaptation in human auditory cortex. *J. Neurophysiol.* **110**, 973–983 (2013).
21. S. Yadav, M. E. Foster, GISE-51: A scalable isolated sound events dataset. *arXiv Prepr. arXiv2103.12306* (2021).
22. A. De Cheveigné, H. Kawahara, YIN, a fundamental frequency estimator for speech and music. *J. Acoust. Soc. Am.* **111**, 1917–1930 (2002).
23. D. M. Green, Detection of auditory sinusoids of uncertain frequency. *J. Acoust. Soc. Am.* **33**, 897–903 (1961).
